# Cobalt-Embedded Metal–Covalent Organic Frameworks for CO_2_ Photoreduction

**DOI:** 10.1021/jacs.4c18450

**Published:** 2025-03-07

**Authors:** Wanpeng Lu, Claudia E. Tait, Gokay Avci, Xian’e Li, Agamemnon E. Crumpton, Paul Shao, Catherine M. Aitchison, Fabien Ceugniet, Yuyun Yao, Mark D. Frogley, Donato Decarolis, Nan Yao, Kim E. Jelfs, Iain McCulloch

**Affiliations:** 1 Chemistry Research Laboratory, University of Oxford, 12 Mansfield Road, Oxford OX1 3TA, U.K.; 2 Department of Chemistry, University of Oxford, Oxford OX1 3QZ, U.K.; 3 Princeton Materials Institute, Princeton University, Princeton, New Jersey 08540, United States; 4 Diamond Light Source, Harwell Science Campus, Oxfordshire OX11 0DE, U.K.; 5 Department of Chemistry, Molecular Sciences Research Hub, Imperial College London, 82 Wood Lane, London W12 0BZ, U.K.; 6 Andlinger Center for Energy and the Environment and Department of Electrical and Computer Engineering, Princeton University, Princeton, New Jersey 08544, United States; 7 Laboratory of Organic Electronics, Department of Science and Technology (ITN), Linköping University Norrköping, Norrköping SE-60174, Sweden

## Abstract

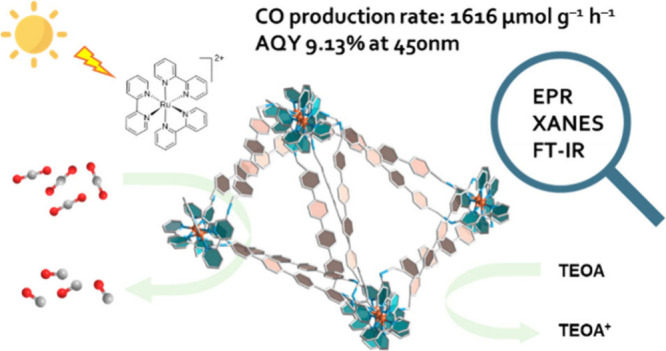

With the pressing urgency to reduce carbon footprint, photocatalytic carbon dioxide reduction has attracted growing attention as a sustainable mitigating option. Considering the important role of catalytic active sites (CASs) in the catalytic processes, control and design of the density and environment of CASs could enhance the catalyst performance. Herein, we report a novel metal–covalent organic framework (MCOF), MCOF-Co-315, featuring earth-abundant Co cocatalysts and conjugation through a covalently bonded backbone. MCOF-Co-315 showed a CO production rate of 1616 μmol g^–1^ h^–1^ utilizing Ru(bpy)_3_Cl_2_ as photosensitizer and triethanolamine (TEOA) as sacrificial electron donor with a 1.5 AM filter, vis mirror module (390–740 nm), and irradiation intensity adjusted to 1 sun and an especially outstanding apparent quantum yield (AQY) of 9.13% at 450 nm. The photocatalytic reaction was studied with electron paramagnetic resonance (EPR) spectroscopy, X-ray absorption near-edge structure (XANES), and in situ synchrotron Fourier Transform Infrared (FT-IR) spectroscopy, and an underlying mechanism is proposed.

Photocatalysis processes, most commonly water splitting and carbon dioxide (CO_2_) reduction, enable solar-to-chemical energy conversion as well as the generation of useful chemicals, which is a promising pathway to ease the energy crisis and reduce the carbon footprint.^1,2^ Anchoring catalytic active sites (CASs) and/or photosensitizers (PSs) onto heterogeneous platforms, such as metal oxides, carbon nitride, metal–organic frameworks (MOFs), or covalent organic frameworks (COFs), has been a widely applied approach to avoid both long distances and random, recombinative intermolecular collisions between CASs and PSs^3−6^ further contributing to higher energy transfer efficiency, enhanced CO_2_ photoreduction activity, and improved material durability.^7,8^ Among these platforms, COFs allow molecular-level design and adjustment of framework functionality, structure diversity, and optoelectronic properties.^9−11^ Various strategies for introducing CASs into COFs include external framework anchoring via chemical bonding or physisorption, attachment of coordination-unsaturated transition metal sites to COF linkers, or integration of heteroatoms in the backbone.^12,13^ Presynthetic metal integration in the backbone or utilizing metal-based molecular complexes as building blocks afforded a new branch of COFs, namely metal–covalent organic frameworks (MCOFs).^14,15^ Through rational material design, MCOFs could simultaneously achieve the following: (i) precise and uniform embedment as well as accurate control of the chemical environment and density of CASs; (ii) new topological possibilities which are challenging with pure organic components; (iii) maintenance of the stability of the covalently bonded backbone to improve material reusability; (iv) preservation of the backbone conjugation to enhance electron transfer rate.

We have developed a novel three-dimensional MCOF material, MCOF-Co-315, from [Co^II^(dabpy)_3_]Cl_2_ [dabpy = (2,2′-bpy)-5,5′-diamine] and [1,1:4,1:4,1-quaterphenyl]-4,4-dicarboxaldehyde through polycondensation. From FT-IR analysis, the formation of imine linkage is evidenced by the intensity increase of the C=N peak at 1613 cm^–1^ (Figure S1),^16^ accompanied by a decrease of the C=O stretch at 1685 cm^–1^ and the amine group peaks around 3300 cm^–1^. From ^13^C nuclear magnetic resonance (ssNMR) spectroscopy, an imine carbon resonance peak at 158 ppm is observed to further confirm the formation of imine linkages (Figure S2).^17^

To construct the MCOF with different net structures compatible with the node and edge building blocks, the Reticular Chemistry Structure Resource (RCSR) was searched.^18^ Within 3D periodic nets, 14 net representations matched the search criteria with six coordination, and one kind of vertex and edge, yet all models resulted in MCOFs with incompatible diffraction patterns to that of the experimental results (Figure S7). Out of 203 plausible 2D periodic nets, only the *hxl* net structure satisfied the search criteria. The *hxl* net with corresponding vertices (nodes) and edges (linkers) was replicated along the *P*6*mm* space group glide lines to explore the offset between 2D layers (Figure S8). Overall, a reductive search strategy allowed us to exclude structures that were inconsistent with the experimental observations. Only the *hxl* MCOF structure (Figure 1a) shows a reasonable agreement with the experimental PXRD pattern (Figure 1b) and the 2D stacking behavior aligns with cryo-transmission electron microscopy (cryo-TEM) results (Figure 1c). Definitive structural determination was inconclusive, hindered by the two-peak experimental PXRD pattern having insufficient data to resolve lattice parameters and the complexity of many node/edge orientation possibilities.

**Figure 1:**
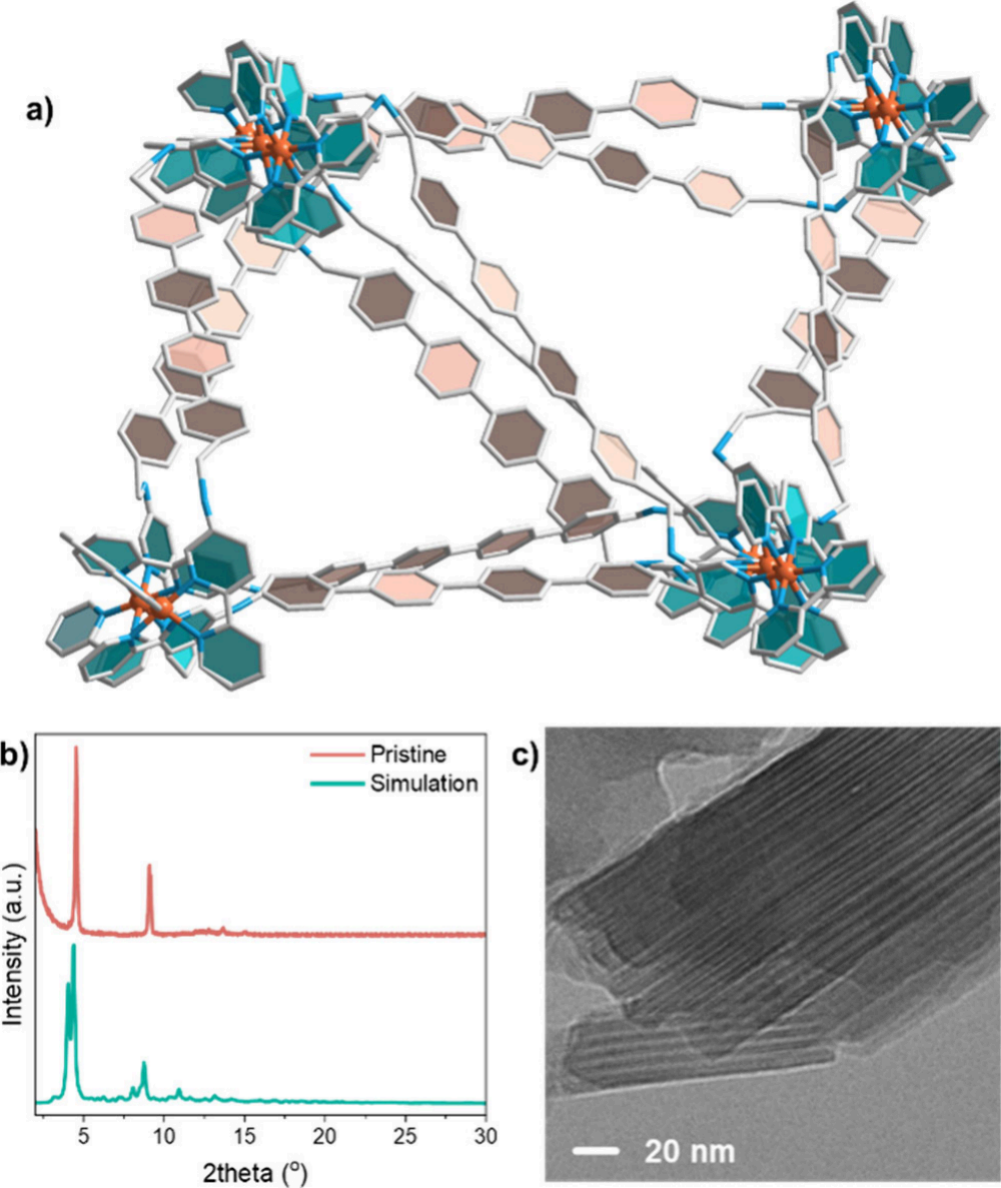
(a) Proposed linkage of MCOF-Co-315, viewed from the *c* axis (Co, orange; N, blue; C, gray; H omitted for clarity). (b) Diffraction pattern obtained from experimental PXRD and simulation. (c) Cryo-TEM of MCO-Co-315 at 20 nm resolution.

At 77 K, MCOF-Co-315 affords a type-I N_2_ adsorption–desorption isotherm, with a BET surface value of 714 m^2^ g^–1^ (Figure 2a). The total pore volume and size obtained from nonlocal density functional theory (NLDFT) are 0.63 cm^3^ g^–1^ and 5.9 Å, respectively (Figure 2b). Thermogravimetric analysis (TGA) of MCOF-Co-315 indicated negligible weight loss before 100 °C and no obvious weight loss is observed with further heating to 270 °C until the MCOF decomposed (Figure S3). From inductively coupled plasma mass spectrometry (ICP-MS), the cobalt concentration in the MCOF was calculated to be 3.89 wt %, comparable to the value calculated from the molecular formula (3.97 wt %). The chemical state of Co was then analyzed by X-ray photoelectron spectroscopy (XPS) (Figure 2c). The 2p_3/2_ and 2p_1/2_ peaks of Co^2+^ are observed at 780.2 and 796.2 eV, accompanied by two satellite peaks at 785.8 and 802.6 eV, respectively.^19^ Based on photoemission spectroscopy in air (PESA), the ionization potential of MCOF-Co-315 is determined as –5.97 eV (Figure 2d). The absorption spectrum of an MCOF suspension in MeCN demonstrates an onset of 532 nm (2.33 eV optical gap) (Figure 2e). An electron affinity of –3.64 eV is therefore estimated (IP + *E*_opt_). The frontier molecular orbital (FMO) energy (−5.97 to −3.64 eV) indicates that MCOF-Co-315 should be suitable for CO_2_ photoconversion to CO (Figure 2f).

**Figure 2:**
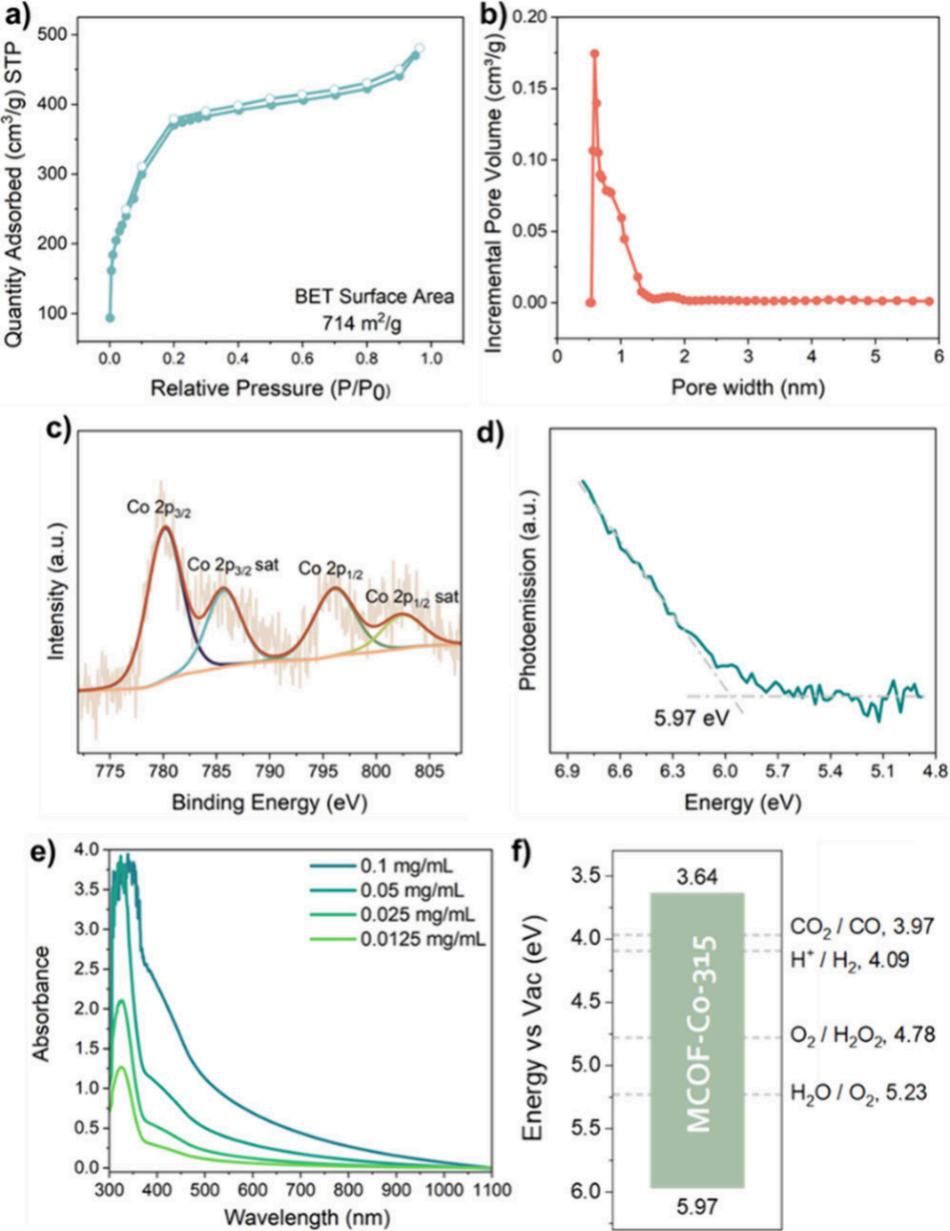
(a) Isothermal N_2_ adsorption–desorption of MCOF-Co-315 at 77 K. (b) Pore size distribution obtained from NLDFT. (c) XPS spectrum for illustrating Co oxidation state in MCOF-Co-315. (d) PESA measurement of drop-cast MCOF-Co-315. (e) UV–vis spectrum of the MCOF-Co-315 suspension in acetonitrile. (f) Calculated FMO energy level.

For the photocatalytic reaction, a 300 W xenon lamp fitted with a 1.5 AM filter and vis (390–740 nm) mirror module was used, with the irradiation intensity adjusted to 1 sun (Figure S9). The reaction was performed in acetonitrile, with Ru(bpy)_3_Cl_2_ as a photosensitizer and triethanolamine (TEOA) as a sacrificial electron donor. The system showed a CO production over the 8 h testing period under visible light yielding 9.9 mmol/g in total (Figure 3a). The calculated CO production rate reached 1616 μmol g^–1^ h^–1^, which is comparable to the highest performing materials reported to date, measured under higher light intensity.^20,21^ Apart from a trace amount of CH_4_ (yield below 0.02 mmol/g after 8 h), no other C product was detected in the gas phase or from ^1^H NMR in the liquid phase. In a series of control experiments, negligible CO was detected in the absence of MCOF, Ru(bpy)_3_Cl_2_, or TEOA, indicating that these species are all crucial for the photocatalytic activity of the system. A ^13^CO_2_ isotope trace experiment was performed to confirm the carbon source of produced CO. The peak at *m*/*z* = 29 from gas chromatography–mass spectrometry (GC-MS) was attributed to ^13^CO, proving that CO originates from CO_2_ reduction (Figure 3b).

**Figure 3:**
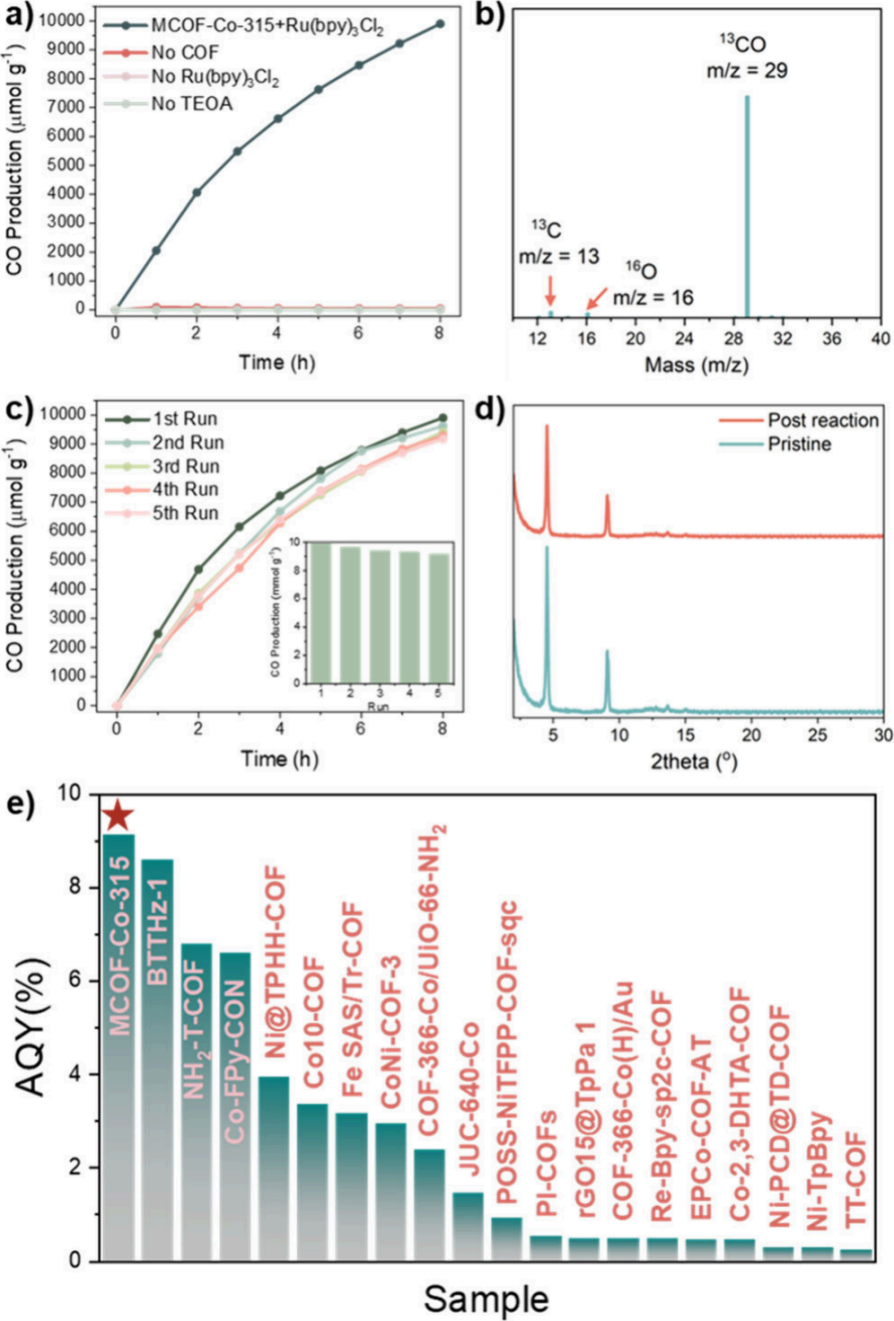
(a) CO production yield over MCOF-Co-315 in 8 h. (b) GCMS of isotope labeling experiment using ^13^CO_2_ as carbon source. (c) Cycle experiment based on the production yield in 8 h using MCOF-Co-315 as photocatalyst. (d) PXRD of MCOF-Co-315 before and after 5 cycles of photocatalysis. (e) AQY comparison of selected COF materials for CO_2_ photoreduction to CO in literature, more details in Table S2.

Subsequently, five consecutive recycling experiments were carried out to evaluate the photocatalytic stability of MCOF-Co-315 (Figure 3c). The MCOF was recollected through centrifugation with photosensitizer and electron donor readded for each cycle. A minor decrease in performance was observed over five cycles, with the yield being 96.0%, 95.1%, 94.5%, and 92.5% of the first run, respectively. PXRD of the postreaction sample revealed retained crystallinity, demonstrating good stability of the compound under catalytic conditions (Figure 3d). At 450 nm, an apparent quantum yield (AQY) of 9.13% was determined for the CO evolution after optimization of the loaded sample amount, which is among the top-performing COF-based catalysts to date (Figure 3e). The wavelength achieving the highest AQY value is consistent with light absorbance over the visible light region (Figure S10). With designed control of cobalt embedment, MCOF-Co-315 demonstrated negligible cobalt leaching (3.85 wt %) during the catalytic reaction, excellent stability and reusability, and a higher production rate than systems featuring postsynthetic cobalt deposition^22^ and an AQY comparable even to organic dye.^23^

Radical species involved in the catalytic process were identified through in situ spin-trapping EPR experiments under photocatalytic conditions.^24^ Measurement of a degassed suspension of MCOF-Co-315, Ru(bpy)_3_Cl_2_, and TEOA in acetonitrile under nitrogen atmosphere with 5,5-dimethyl-1-pyrroline-N-oxide (DMPO) added showed no signals in the dark or under illumination in the absence of CO_2_ (Figure 4a). Addition of CO_2_ to the same sample preilluminated under a nitrogen atmosphere led to immediate appearance of a signal attributed to the DMPO–CO_2_^•–^ adduct (*g* = 2.0054 and hyperfine coupling constants *A*^N^ = 14.8 G and *A*^H^ = 18.1 G).^25^ When CO_2_ was added to the degassed solution before illumination, a gradual growth of the DMPO–CO_2_^•–^ adduct was observed as a function of time during illumination (Figure 4a,b). This supports the presence of activated [Ru(bpy)_3_]^2+^* within the system after illumination, which in the presence of TEOA forms the highly reducing [Ru(bpy)_3_]^+^, followed by electron transfer to MCOF-Co-315 in the presence of CO_2_, completing the photocatalytic cycle and leading to the formation of a DMPO–CO_2_^•–^ adduct.

**Figure 4:**
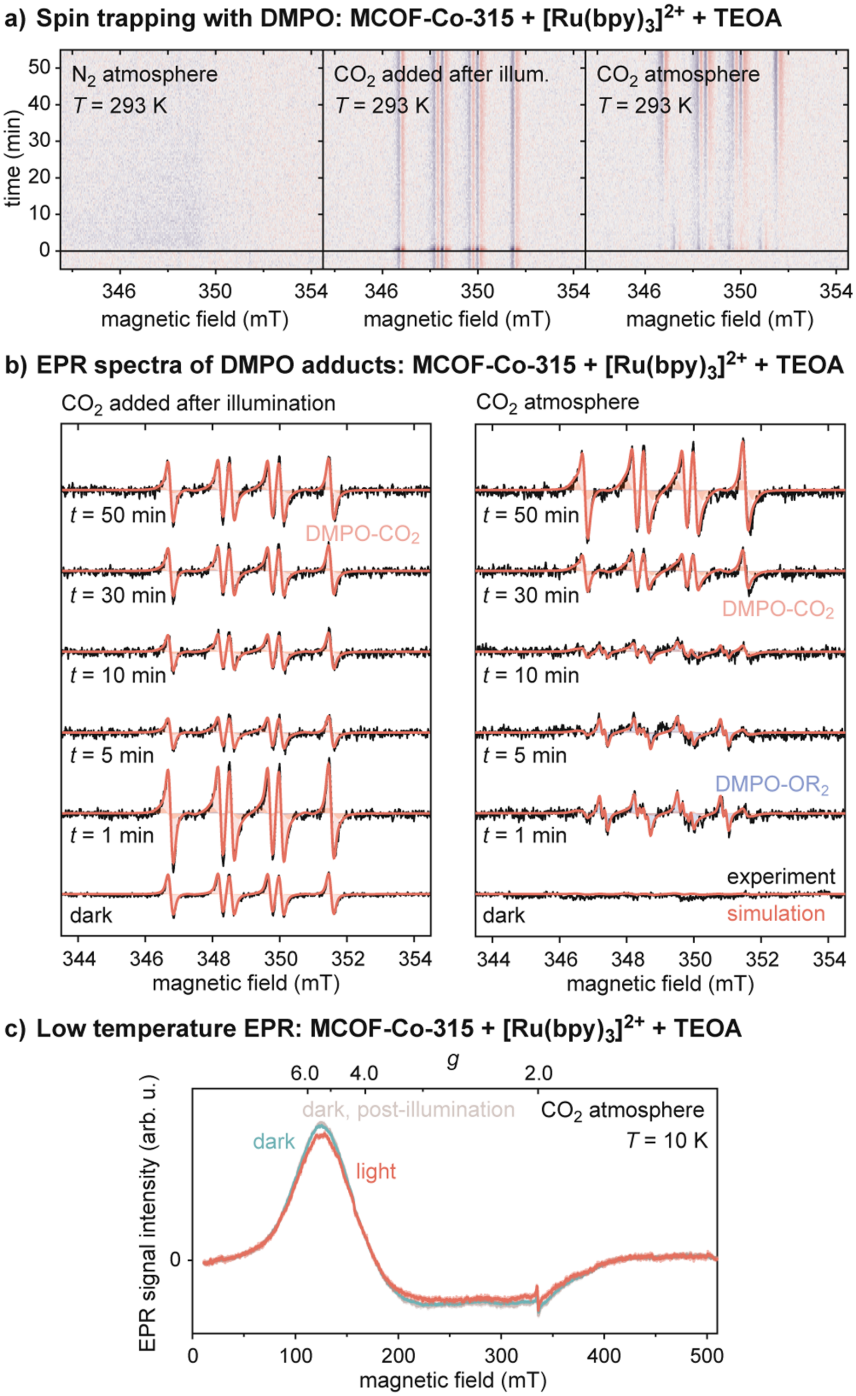
(a, b) In situ X-band EPR measurements performed as a function of time during photocatalysis with DMPO as a spin trap compared to simulations of DMPO adduct spectra. (c) EPR spectra of MCOF-Co-315 with Ru(bpy)_3_Cl_2_ before, during, and after illumination at 10 K.

To characterize the oxidation state of Co, additional EPR measurements were performed at cryogenic temperatures on CO_2_-saturated solutions containing MCOF-Co-315, Ru(bpy)_3_Cl_2_, and TEOA (Figure 4c). For MCOF-Co-315, a broad signal extending from *g* = 6 to *g* = 2 was observed, in addition to a very narrow weak signal at *g* ≈ 2. The broad signal is attributed to high-spin CoII andcould be reproduced by simulations assuming an effective *S* = 1/2 system with *g*_eff,*x*_ = 5.04, *g*_eff,*y*_ = 3.59, and *g*_eff,*z*_ = 1.95 with significant *g*-strain to reproduce the extensive broadening (see SI section 6.2 for details). In situ illumination led to an immediate decrease in signal intensity to about 85% of the initial intensity, with no further changes for prolonged illumination. The decrease in the intensity of the broad EPR signal is reversible, with the signal returning to the initial intensity in the dark, suggesting a reversible transformation from Co^II^ to diamagnetic Co^III^.

Employing in situ synchrotron FT-IR spectroscopy, with increments of CO_2_ concentration, a gradual increase of peak intensity for the antisymmetric stretch of physically adsorbed CO_2_ molecules was observed at 2335 cm^–1^ (Figure 5a).^26^ Noteworthy, an emerging peak at 655 cm^–1^ was also observed, which can be assigned to Co–O interaction between Co active sites and CO_2_.^27^ XANES was performed to further investigate the chemical state of cobalt before and after the photocatalytic reaction. XANES analysis at the Co K-edge (Figure 5b) revealed that the absorption edge of MCOF-Co-315 shifted to higher energy after the reaction. This shift indicates a slight increase in the average valence state of Co atoms postreaction,^28^ consistent with EPR and further analysis from extended X-ray absorption fine structure (EXAFS) (Figure S18).

**Figure 5:**
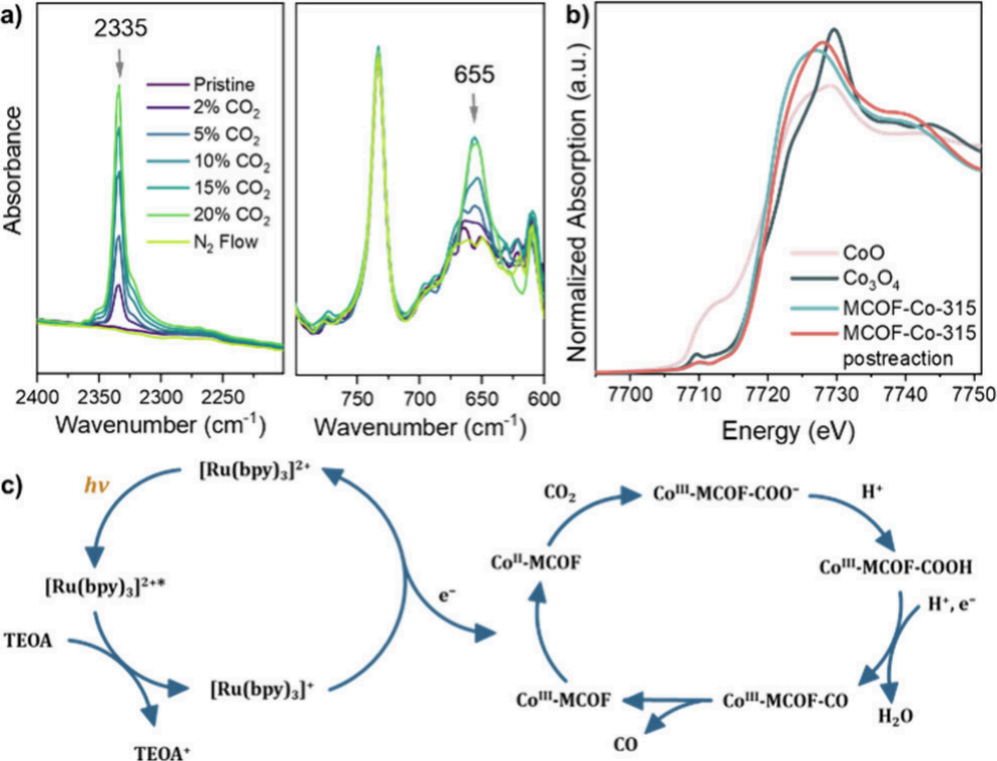
(a) In situ synchrotron FT-IR spectroscopy of MCOF-Co-315 with incremental CO_2_ concentration in gas stream. (b) Normalized Co K-edge XANES spectra of MCOF-Co-315, MCOF-Co-315_post reaction, and reference samples (CoO and Co_3_O_4_). (c) Proposed reaction mechanism.

A plausible reaction mechanism in accordance with these observations is proposed, as outlined in Figure 5c. Initially, Co^II^ in MCOF-315 undergoes oxidation by CO_2_, producing Co^III^ and generating the radical *CO_2_^–^. Subsequently, *CO_2_^–^ is converted into the adsorbed *CO intermediate, which then desorbs from the Co^III^ active site, forming free molecular CO. The Co^III^ species then acquires an electron from the light-activated photosensitizer ([Ru(bpy)_3_]^+^), regenerating the Co^II^ site, thereby facilitating the continuation of the catalytic cycle.

In conclusion, we developed a novel MCOF material, embedded with earth abundant transition metal cobalt as the active site, demonstrating exceptional CO_2_ reduction performance and state of the art AQY. As a 2 e^–^ transfer process, photoreduction of CO_2_ to CO is assisted by the interaction between the CO_2_ molecule and metal sites, leading to a change of oxidation state of the metal. While the solar-to-chemical conversion efficiency of MCOF-Co-315 is promising for carbon capture, utilization and storage (CCUS), its limited production rate necessitates further optimization. This work offers new insights for catalyst development, especially for the embedment control of cocatalysts for solar-driven CO_2_ reduction.

## Supporting Information Available

Additional experimental details, materials, and methods, including photographs of experimental setup (PDF)
