# Supplementary material for: Cobalt-Embedded Metal–Covalent Organic Frameworks for CO2 Photoreduction
Source: J Am Chem Soc. 2025 Mar 7;147(11):9056–61. doi: 10.1021/jacs.4c18450 (PMC11926853; doi:10.1021/jacs.4c18450)
Supplement: Supplementary file 1 — ja4c18450_si_001.pdf [file ja4c18450_si_001.pdf]

Supporting Information

Cobalt-Embedded Metal-Covalent Organic Frameworks for CO_2 Photoreduction

Wanpeng Lu[1], Claudia E. Tait[2], Gokay Avci[5], Xian'e Li[1,7], Agamemnon E. Crumpton[1], Paul Shao[3], Catherine M. Aitchison[7], Fabien Ceugniet[1], Yuyun Yao[1], Mark D. Frogley[4], Donato Decarolis[4], Nan Yao[3], Kim E. Jelfs[5], Iain McCulloch[1,6*]

1. Chemistry Research Laboratory, University of Oxford, 12 Mansfield Road, Oxford, OX1 3TA, UK.

2. Department of Chemistry, University of Oxford, Oxford, OX1 3QZ, UK.

3. Princeton Materials Institute, Princeton University, Princeton, New Jersey 08540, USA.

4. Diamond Light Source, Harwell Science Campus, Oxfordshire, OX11 0DE, UK.

5. Department of Chemistry, Molecular Sciences Research Hub, Imperial College London, 82 Wood Lane, London, W12 0BZ.

6. Andlinger Center for Energy and the Environment and Department of Electrical and Computer Engineering, Princeton University, Princeton, NJ, 08544, USA.

7. Laboratory of Organic Electronics, Department of Science and Technology (ITN), Linköping University, Norrköping SE-60174, Sweden.

Contents

1. Materials and General Characterization

2. MCOF Synthesis

3. Structural Simulation

4. Inductively Coupled Plasma Mass Spectrometry Residual Pd Measurements (ICP-MS)

5. Photocatalysis

6. Electron Paramagnetic Resonance

7. Synchrotron FTIR Micro-spectroscopy

8. Catalytic Performance in Literature

9. Single Crystal X-Ray Diffraction of $[Co^{II}(dabpy)_3]Cl_2$

10. Extended X-ray Absorption Fine Structure Analysis

11. References

1. Materials and General Characterization

All the reagents were used as received from commercial suppliers without further purification. Powder X-ray diffraction (PXRD) patterns were collected using a Bruker D8 Advance Eco diffractometer (40kV and 30 mA) using Cu Kα radiation (λ = 1.5406 Å). TEM was obtained on Titan Krios G3 cryo Transmission Electron Microscope (cryo-TEM) at 300kV. Ultraviolet-visible (UV-vis) spectra were recorded on a UV-vis spectrophotometer (Shimadzu UV-1800). Photoemission spectroscopy in air (PESA) spectra used a KP Technology APS02 system over wavelengths of ca. 180-280 nm. Material films were obtained from drop casting DMF suspension onto ITO. The intercept of the PESA signal to the baseline was used to estimate the ionisation potential (IP) of the MCOF. The optical bandgaps were estimated using the intercept of the absorption to the baseline from the UV-vis spectra. Subtracting the optical bandgap from the ionization potential energy was taken as an estimate of electron affinity (EA). N_2 adsorption isotherms were measured on an Anton Paar Autosorb iQ-XR gas sorption analyser instrument at 77 K, where the sample was heated at 120 °C for 8 h under vacuum for activation. TGA was measured on a Q20-2025 from TA instruments under air flow. X-ray photoelectron spectroscopy (XPS) spectra were recorded using a Scienta-200 hemispherical analyzer equipped with a monochromatized Al Kα radiation source (photon energy, Eph = 1486.6 eV). The instrument was calibrated by referencing the Fermi level and the Au 4f7/2 peak position of a gold foil cleaned via Ar^+ ion sputtering. All measurements were carried out under ultra-high vacuum conditions, with a base pressure maintained below 10^{-9} mbar. X-ray absorption near edge structure (XANES) was measured at the B18 beamline at Diamond Light Source. The data were collected at the Co K edge (7.708 keV) on Si (111) using a Pt Mirror Coating in transmission mode. XANES data were energy calibrated using the Co metallic foil and normalized with the Athena software.

2. MCOF Synthesis

The building block $[Co^{II}(dabpy)_3]Cl_2$ [dabpy = (2,2'-bpy)-5,5'-diamine] was synthesised as reported in the literature.[1] A mixture of $CoCl_2{\cdot}6H_2O$ (0.69 g) and 2,2′-bipyridine-5,5'-diamine (1.6 g) was dissolved in ethanol (100 ml) and refluxed for 2 hours under N_2. The product was separated with filtration and characterised with HR-MS. HRMS (ESI^+, CH_3OH) calculated for $C_{30}CoH_{30}N_6C_{l2}$, 604.4386 ($[M+H]^+$); found, 604.4472. Yield = 73% (base on Co).

A Pyrex glass tube (10 mL) was charged with $[Co^{II}(dabpy)_3]Cl_2$ (17.2 mg, 0.03 mmol), [1,1:4,1:4,1-Quaterphenyl]-4,4-dicarboxaldehyde (32.6 mg, 0.09 mmol), 0.3 mL benzylic alcohol, 0.7 mL mesitylene and 0.1 mL 6 M acetic acid aqueous solution. The tube was first sonicated for 30 minutes and then flash frozen at 77 K (liquid N_2 bath) and degassed over three freeze-pump-thaw cycles. The internal pressure was evacuated to 10^{-3} mbar. The tube was sealed and placed in a preheated oven at 120 °C for 3 days. After heating, the tube was cooled down and cut. The formed light-yellow precipitate was filtered and washed with water and methanol several times. The product was then purified via methanol Soxhlet extraction. Finally, the powder was dried in a normal oven at 80 °C. Yield = 80.3 % (40 mg).

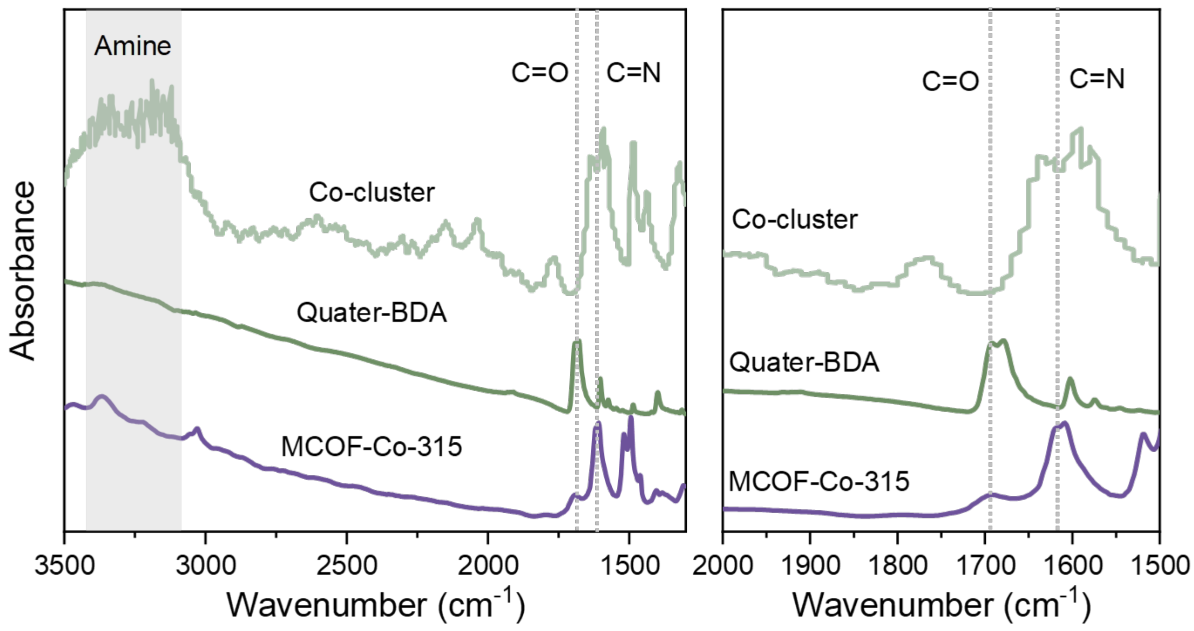

Fig.S1. FT-IR of building blocks and pristine MCOF material.

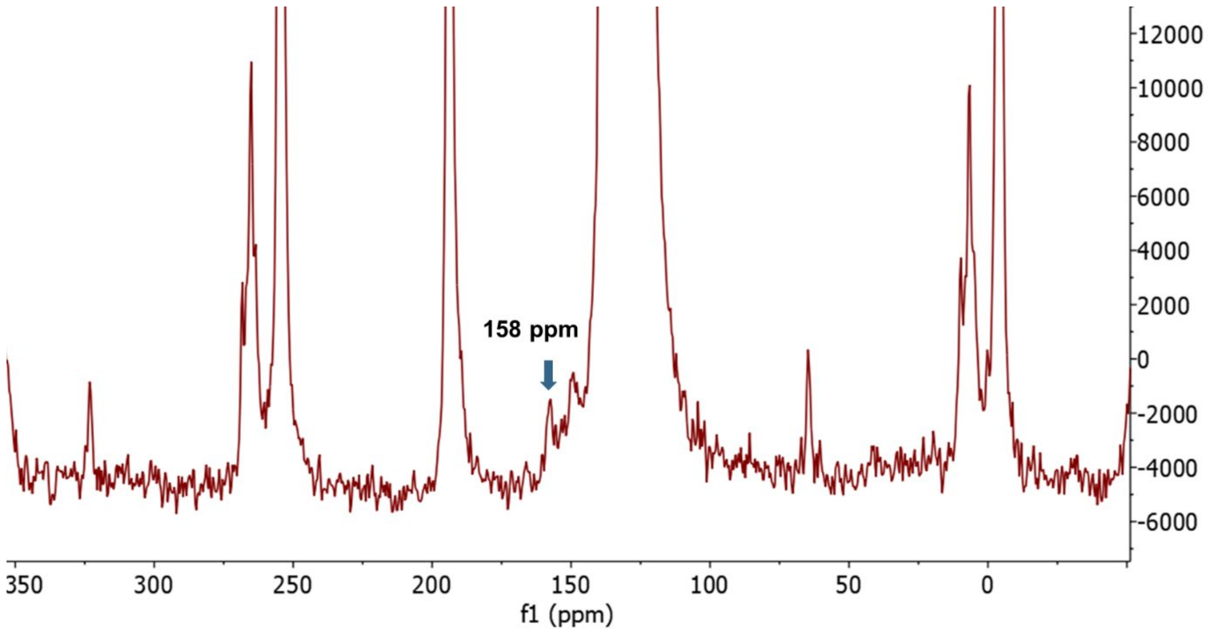

Fig.S2. The solid-state ^{13}C NMR spectrum of MCOF-Co-315.

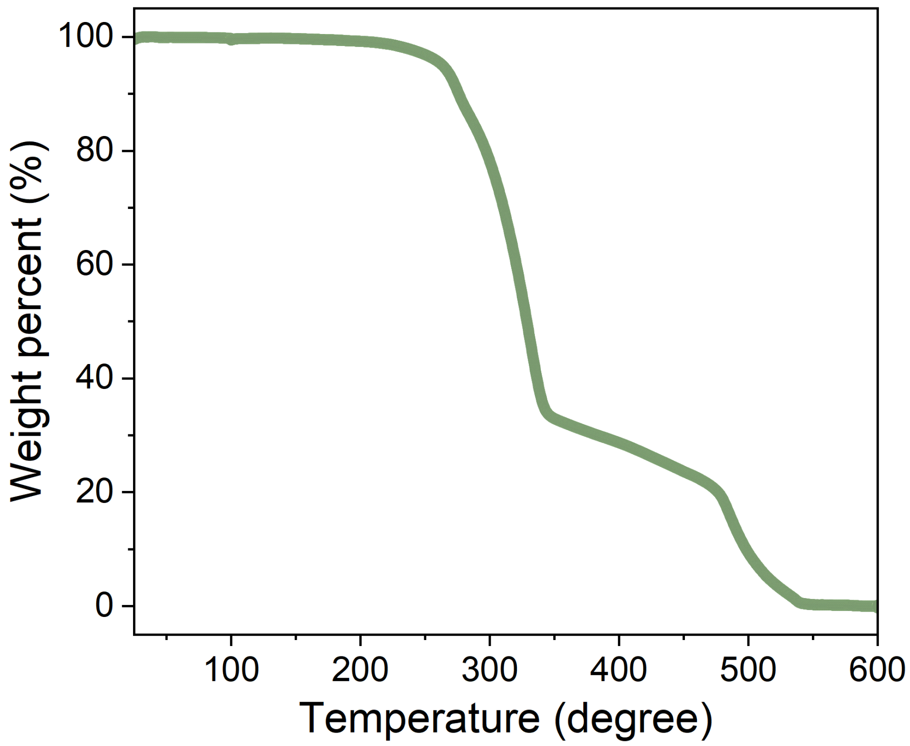

Fig.S3. Thermogravimetric analysis of MCOF-Co-315.

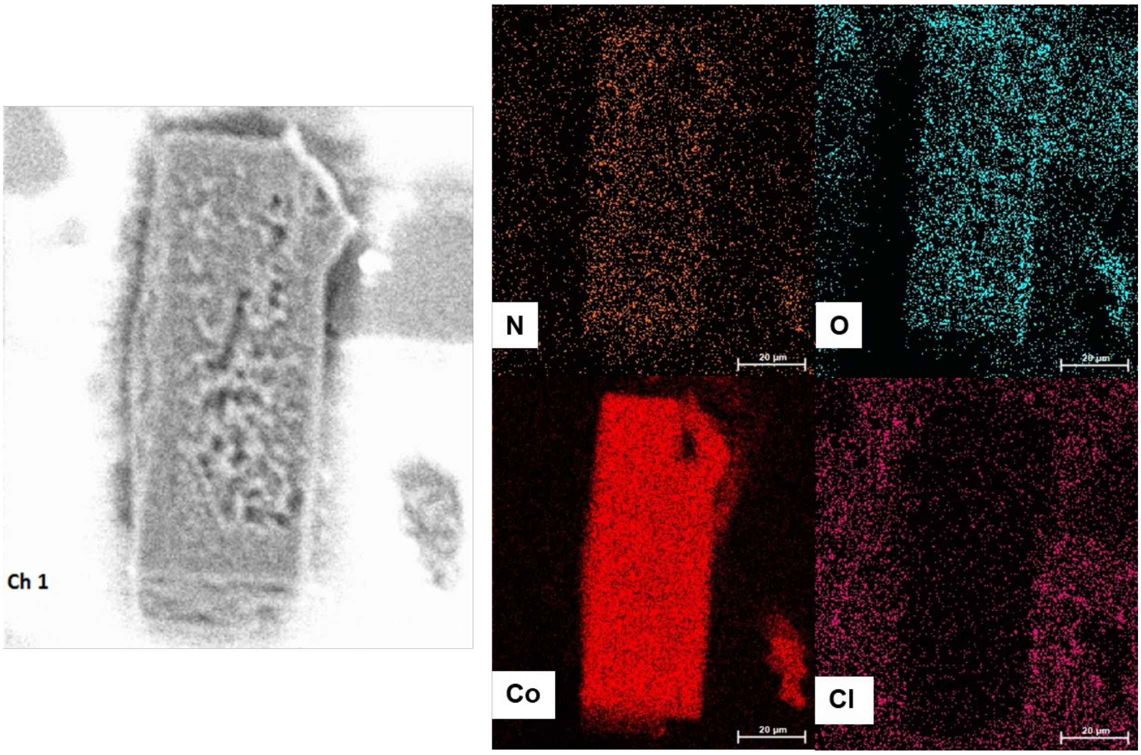

Fig.S4. Energy-dispersive X-ray spectroscopy from scanning electron microscope (SEM) for element distribution in MCOF-Co-315.

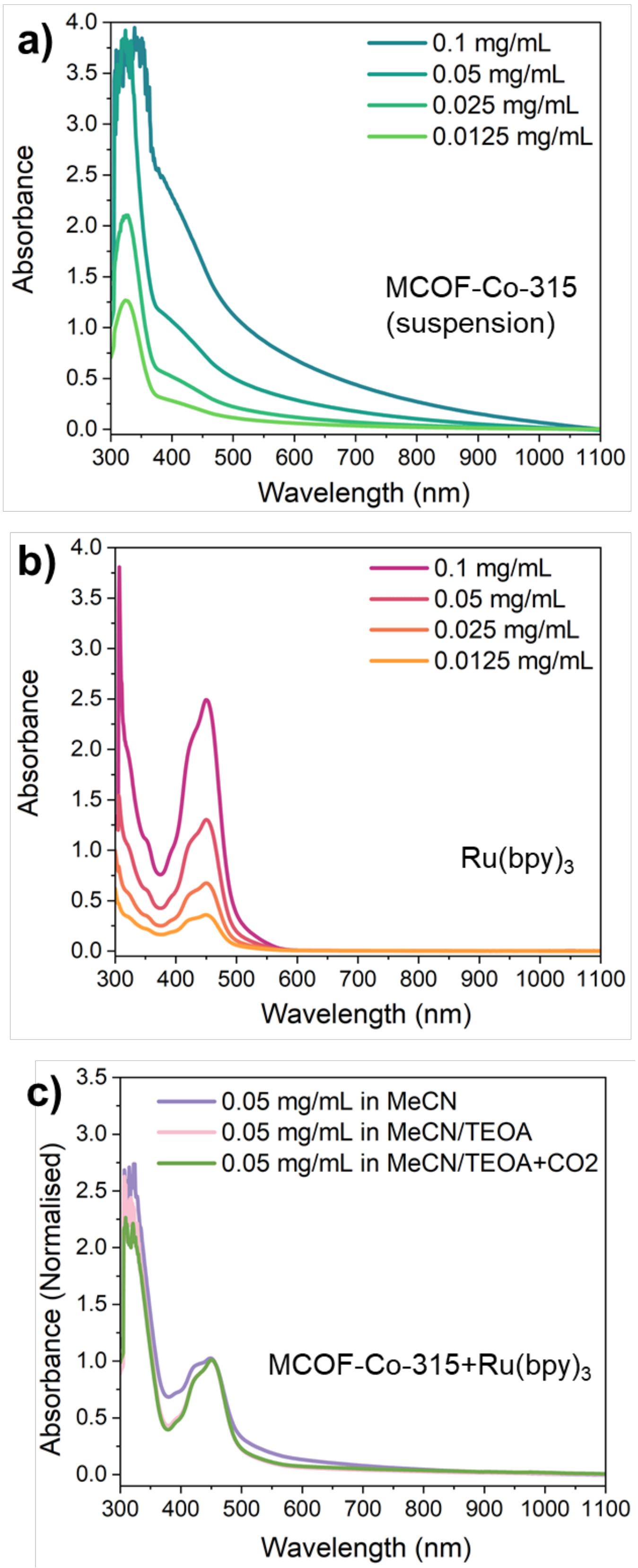

Fig.S5. UV-vis spectra of **a)** MCOF-Co-315 suspension in MeCN; **b)** Ru(bpy)$_3$ solution in MeCN; c) MCOF-Co-315 and Ru(bpy)$_3$ suspension under different conditions.

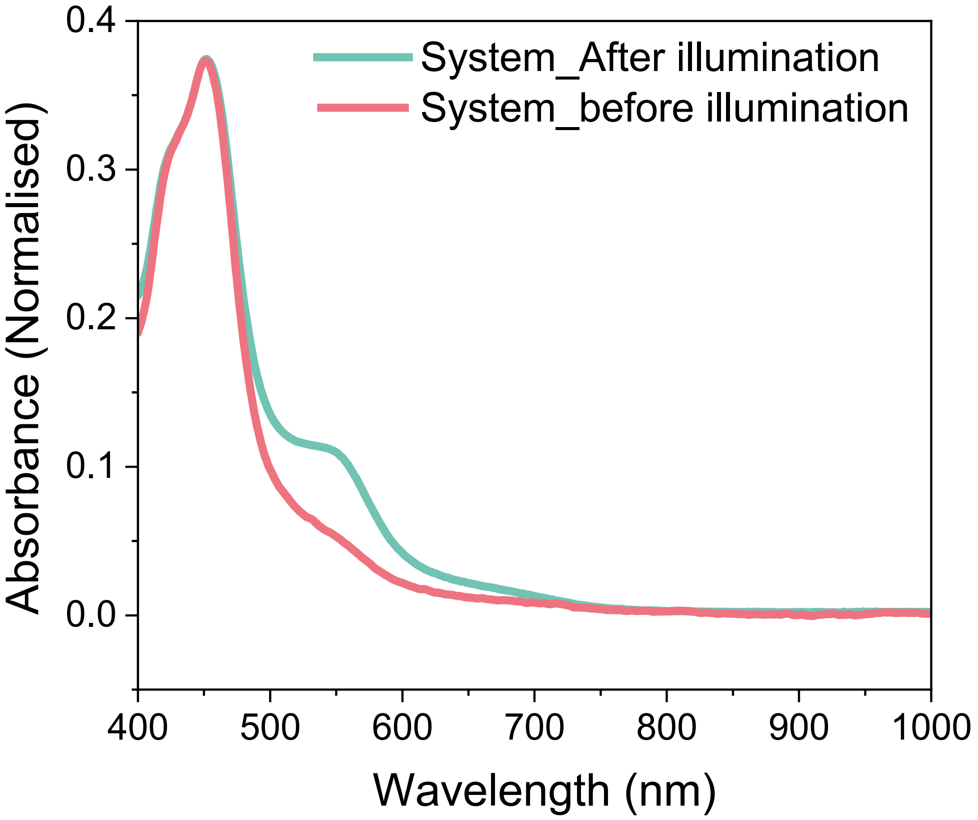

Fig.S6. UV-vis spectra of MCOF-Co-315 and $Ru(bpy)_3$ suspension in MeCN with TEOA under CO_2 atmosphere before and after 8 hour illumination.

3. Structural Simulation

The Topologically Based Crystal Constructor (ToBaCCo) software version 3.0 without any modifications was used to constructed M-COF representations.[3] The edge building block was constructed with *stk*[4] from the SMILES representation, and the node building block was adapted from a Ru-based structure[5] where Ru atoms were exchanged with Co atoms with Mercury.[6] Within 3D periodic nets, 14 net representations matched the search criteria with six coordination, one kind of vertex and edge (*acs, bcs, crs, fdt, foo, hxg, lcy, pcu, pcu-z, pok, qln, qlo, qok* and *rok*, respectively, where *acs*, *bcs*, *crs*, *hxg*, *lcy* and *pcu* nets were present in ToBaCCo, RCSR data archive June 2019) yet all of the net embeddings resulted in M-COFs with incompatible diffraction patterns, to that of the experimental results, with differing numbers of peaks (**Fig.S7**). Out of 203 deposited 2D periodic nets, only the *hxl* net structure satisfied the search criteria. (**Fig.S8**) The *hxl* net with corresponding vertices (nodes) and edges (linkers) was replicated along the *P6mm* space group glide lines, to explore the offset between layers. To account for position adaptation due to metal change, Density Functional Theory (DFT) calculations with the CP2K software package[7] were employed using the PBE functional,[8] def2-TZVP basis set,[9] a grid size of 400 Ry and a relative cut-off value of 100 Ry, while considering any spin polarisation of unpaired electrons. Dummy atoms were used to connect the edge and node building blocks. The resultant structures, and their lattice parameters were reoptimized with L-BFGS-B algorithm and UFF force field, as implemented within ToBaCCo software (which does not achieve a full optimization). It should be noted that our search was within the bounds of known/observed net embeddings in Reticular Chemistry Structure Resource, where net embeddings beyond deposited ones were not explored. Our *de novo* construction methodology only checks for basic catenation, where higher order catenation (up to 3-fold) was only searched for *hxl* net embedding.

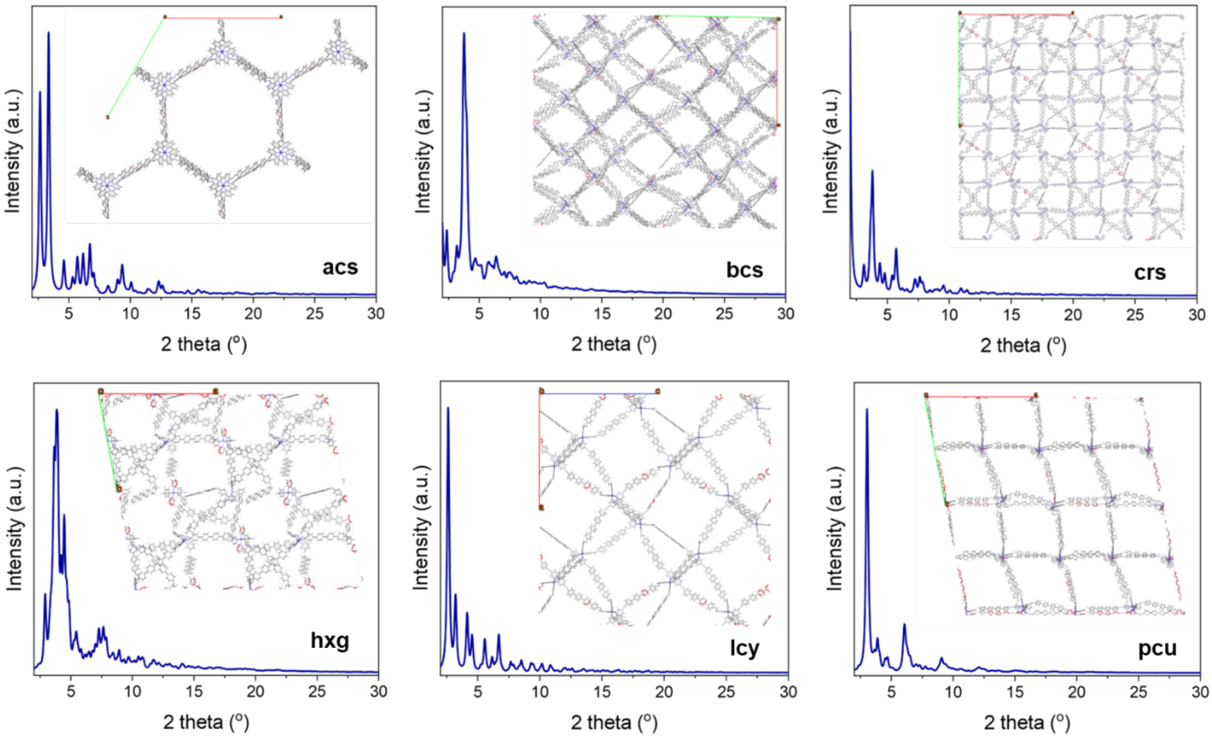

Fig.S7. Ilustration of computational and experimental PXRD mismatch between constructed 3D MCOFs from a-e) *acs*, *bcs*, *crs*, *hxg*, *lcy* and *pcu* net embeddings, respectively.

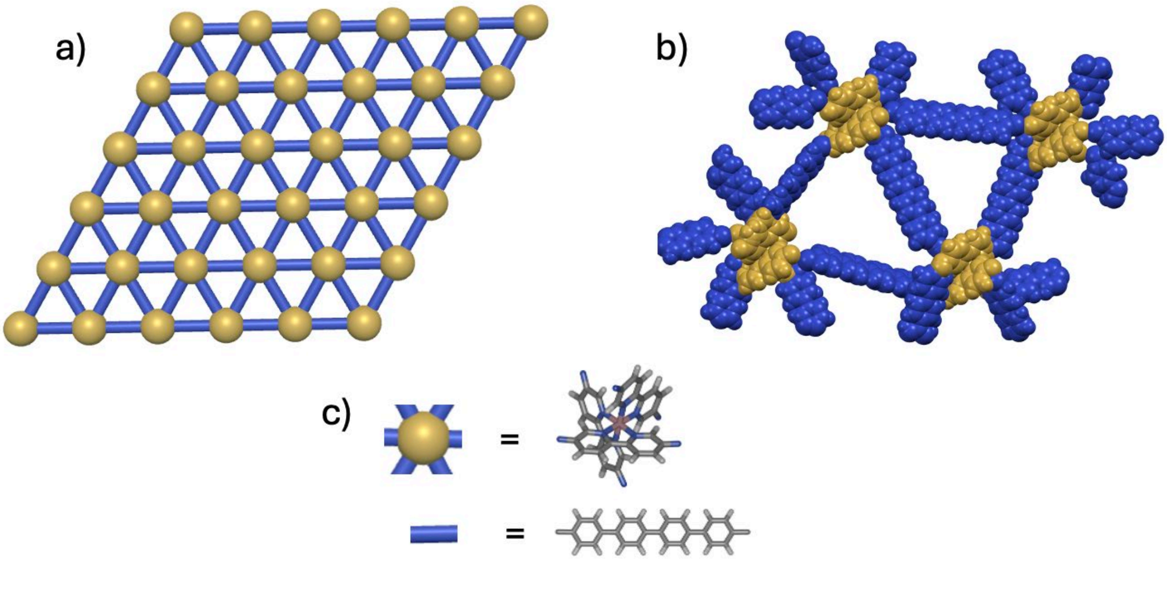

Fig.S8. a) Ball and stick representation of the hxl net topology. b) Illustration of corresponding MCOF node and linkers placed on net topology embeddings. c) The building blocks are connected via introducing dummy atoms between end points.

4. Inductively Coupled Plasma Mass Spectrometry Residual Pd Measurements (ICP-MS)

2 mg polymer was digested in 2 mL nitric acid using an Anton Paar Microwave Digestive System Multiwave GO Plus for 2 h. The digested polymer acidic solution was diluted to a total volume of 100 mL with deionised water. Inductively coupled plasma mass spectrometry was conducted using a Shimadzu ICPMS-2030 with 5 calibration solutions between 0 and 500 ppb cobalt.

5. Photocatalysis

Photocatalysis measurements were conducted in a quartz reactor connected in flow with a custom Agilent 8890 GC System. 5 mg of MCOF is loaded into the reactor with 5 mg of $[Ru(bpy)_3]Cl_2 \cdot 6H_2O$, 5 mL of TEOA and 30 mL of MeCN. The reactor was degassed thoroughly before analysis and backfilled with CO_2 to a pressure of 700-900 mbar. Gas products in the headspace were circulated using a solenoid pump and were sampled once per hour by automatic injection. Samples were cooled by a flow of water at 15°C around the reactor. Samples were illuminated from above using an Asahi Max-303 300W Xenon Light Source fitted with a UV-IR mirror module, an AM 1.5 G filter, a quartz light guide and a (1.0x) collimator lens. The intensity of light was adjusted to 1 sun using a Newport calibrated reference cell. The EQE was calculated by dividing 2 x the number of moles of CO evolved per hour by the total number of photons incident on the sample surface (illumination area = 4.4 ± 0.1 cm^2) per hour. Photon flux at the height of the sample was measured using a calibrated ThorLabs probe.

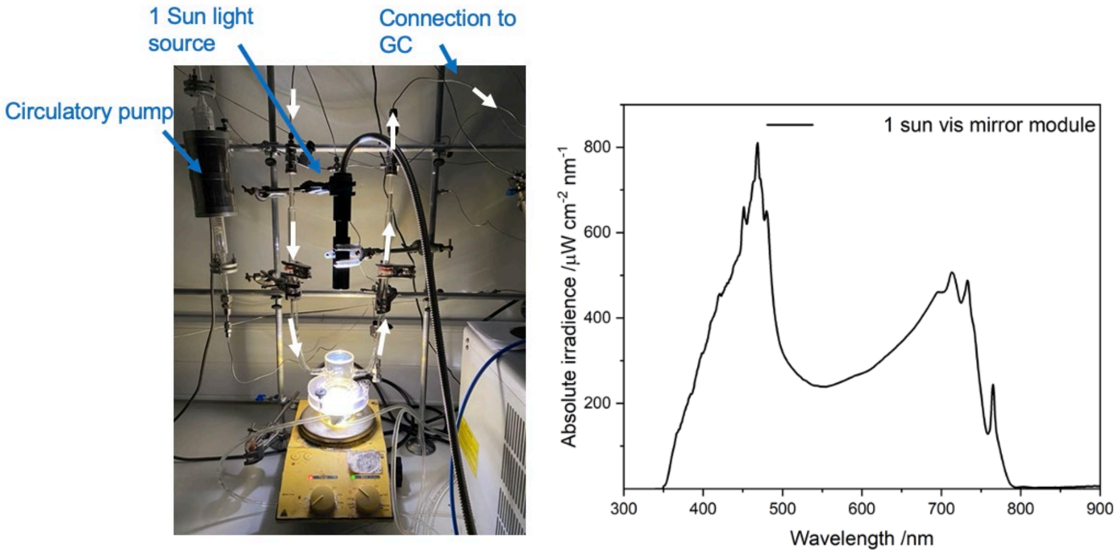

Fig.S9. Left: photocatalytic performance testing setup. Right: spectral output of the light source used in photocatalysis experiments.

The AQY of MCOF-Co-315 was measured with 10 mg of MCOF, 10 mg of $[Ru(bpy)_3]Cl_2 \cdot 6H_2O$, 5 mL of TEOA and 30 mL of MeCN. The test was carried out in the same manner as described above, except it was irradiated using a filter to monochromatize the light source centred at 450 nm, 550 nm and 650 nm, respectively. Using a Thorlabs light intensity probe set to different wavelegth, the illumination power was measured at the same position the sample sits during testing. The light intensity received by the sample per unit area was then calculated from the measured incident power and the area of light intensity detector aperture. Given that the illuminated photocatalysis cell was 15.9 cm^2, the power incident on the photocatalyst was then calculated. The photon flux incident on the sample was then calculated according to photon energy of different wavelength. Dividing by Avogadro's constant to get the incident flux of photons in moles. The photocatalyst test was conducted for 8 h, and the CO production rate was calculated between 4 and 8 h. Given two photons are required to produce 1 mole of photons, the apparent quantum yield was calculated using the following equation:

$$AQY\ \% = \frac{2\, n_{\mathrm{CO}\ produced}}{n_{incident\ photons}} \times 100$$

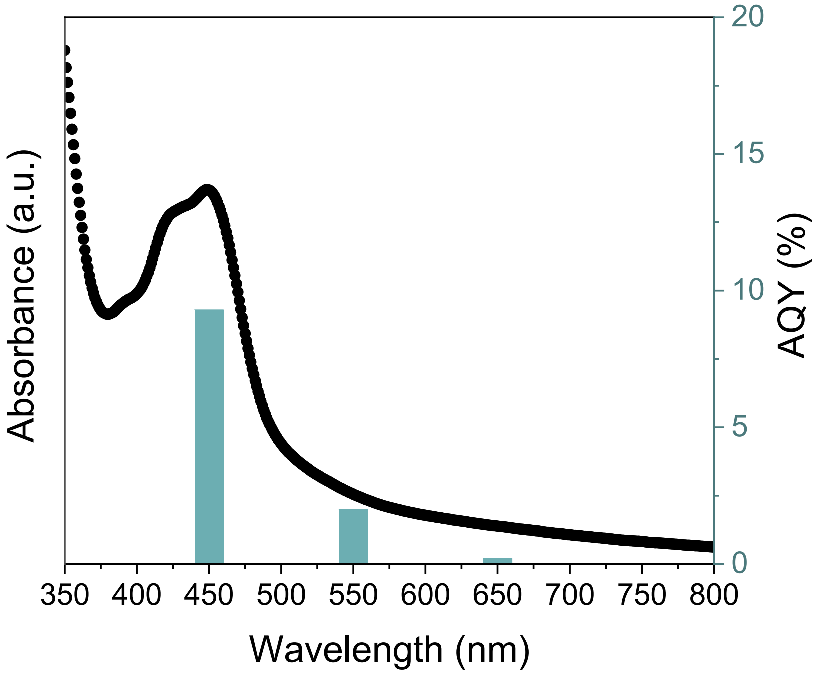

Fig.S10. AQY values under different illumination wavelengths compared to the UV-vis absorption spectrum.

6. Electron Paramagnetic Resonance

6.1 Experimental details

The EPR spin trapping experiments were performed on acetonitrile solutions transferred to 1.0 mm ID EPR tubes. 1µL DMPO (Dojindo) was added to the solution immediately prior to the EPR measurements and the measurements were all performed under inert or CO_2-saturated atmosphere. The measurements under nitrogen atmosphere were performed on solutions degassed by several freeze-pump-thaw cycles, DMPO was added inside a glovebox immediately prior to the measurement. Measurements were also performed on the same samples after adding CO_2 to the sample in the EPR tube using a gas-filled balloon.

The continuous-wave EPR experiments were performed on a Bruker EMXmicro spectrometer equipped with a Premium bridge. The sample was centred vertically with standard PTFE supports in the cylindrical TE011 mode of a super high sensitivity Bruker probe head (ER4123-SHQE-W1) at room temperature. EPR measurements were performed in the dark and under in-situ illumination through the optical window of the resonator (optical grid with eight 2x5 mm slits over a height of 23 mm) with a Schott KL 2500 LED cold light source with a liquid light guide (power setting 30%, lamp maximum 1100 lumen). The EPR spectra were recorded as a function of time at a microwave frequency of around 9.8 GHz using a microwave power of 2 mW and a modulation amplitude of 0.1 mT for the measurements in the presence of DMPO and of 1 mT for the measurements of just the solution with the photocatalyst. A field calibration was performed with a standard solid N@C60 sample with a known g-value.[10] Spectral simulations were performed using the EasySpin toolbox (version 6.0.1).[11]

For quantitative measurements, the simulations of the individual contributions to the EPR spectra at different times were used to estimate the corresponding number of spins from the double integral using the recorded *Q*-value and the resonator calibration parameter set provided by the manufacturer.

Low temperature EPR experiments were performed at 10 K on MCOF-Co-315 in the presence of $[Ru(bpy)_3]^{2+}$ and TEOA in a 1:1 acetonitrile:toluene mixture flash-frozen in liquid nitrogen prior to insertion into the Oxford Instruments ESR900 continuous flow helium cryostat used for the EPR measurements at cryogenic temperatures. Measurements were performed on ca. 60 µl of solution in 3.0 mm ID quartz EPR tubes. Background measurements were performed on a sample containing only the solvent mixture and used for background correction of the

experimental data. In situ illumination of the sample followed the same approach as for the room temperature measurements.

6.2 Detection of short-lived photoinduced radicals by spin-trapping

The generation of short-lived radicals formed during the photoreduction cycle of the MCOF-Co-315 photocatalyst was investigated by EPR measurements performed on solutions containing the spin trap DMPO. Reaction of short-lived radicals with an initially diamagnetic DMPO molecule results in formation of long-lived paramagnetic adducts with spectral parameters determined by the type of trapped radical. The appearance of the EPR spectra of the DMPO adducts is determined by the strength of the electron-nuclear hyperfine coupling to the nitrogen nucleus and to the β proton (as well as in some cases additional protons on the trapped radical). The parameters for the different types of DMPO adducts observed experimentally were determined by simulation of the experimental data and are summarised in **Table S1** and shown in **Fig.S11**. Assignment of the different signals to specific DMPO adducts was attempted based on comparison to literature values.[12]

The EPR measurements performed on degassed suspensions of MCOF-Co-315, $Ru(bpy)_3Cl_2$ and TEOA in acetonitrile in the presence of DMPO discussed in the main text include signals of different DMPO adducts: the DMPO-CO_2^- adduct, another adduct with hyperfine parameters characteristic of a carbon-centred radical (DMPO-CR) and two adducts with oxygen-centred radicals (DMPO-OR_1, DMPO-OR_2). Simulation of the EPR spectra recorded as a function of time prior to and during illumination as a linear combination of the different adduct spectra yields the evolution of their concentration in time as shown in **Fig.S12**.

Reference measurements performed in the absence of $[Ru(bpy)_3]^{2+}$, in the absence of the scavenger TEOA and on just TEOA in acetonitrile are shown in **Fig.S13** and also exhibit contributions from DMPO adducts, DMPO-CR and DMPO-OR_1 in the absence of $[Ru(bpy)_3]^{2+}$ and for TEOA only, and additional adducts attributed to degraded DMPO in the absence of TEOA. The DMPO-OR_2 radical adduct is formed at high concentrations immediately after illumination exclusively in the presence of $[Ru(bpy)_3]^{2+}$ and then disappears within a couple of minutes.

The simulation parameters for DMPO-CO_2^- and DMPO-CR are quite similar, and both are in agreement with DMPO adducts of carbon-centred radicals.[12] The sharper signal that was found to grow in after illumination of CO_2-saturated solutions was assigned to the DMPO-CO_2^-

adduct. Due to the similarity of the spectra for the two adducts, there is some level of uncertainty in the concentration of each, although in almost all cases simulations taking both contributions into account were required to achieve satisfactory agreement between experiment and simulation. The simulation parameters for DMPO-OR_1 and DMPO-OR_2 are also similar and indicate adducts involving an oxygen-centred radical, both types of adducts appear to be present in solutions exposed to air and therefore containing oxygen. The DMPO-OR_2 adduct is characterised by resolved hyperfine coupling to an additional hydrogen on the trapped radical and is present at high concentrations immediately after the onset of illumination in solutions containing both $[Ru(bpy)_3]^{2+}$ and oxygen. The DMPO-OR_1 adduct is characterised by a broader linewidth and therefore no resolved additional hyperfine couplings. DMPO-CR and DMPO-OR_1 both exist in control experiment on just TEOA in acetonitrile, and their intensity increases under illumination (**Fig.S13**).

EPR measurements performed on MCOF-Co-315, $Ru(bpy)_3Cl_2$ and TEOA in acetonitrile in the absence of the DMPO spin trap both under nitrogen atmosphere and for a CO_2-saturated solution are shown in **Fig.S14** (after subtraction of the background signal recorded under nitrogen atmosphere in the dark). Under nitrogen atmosphere, illumination leads to the appearance of two EPR signals: a broad signal centred at a *g*-value of 1.9927 (peak-to-peak linewidth of 7 mT, Lorentzian) and a narrower signal centred at a *g*-value of 2.0006 (peak-to-peak linewidth of 1.5 mT, Gaussian). These signal contributions are attributed to photoinduced charged states on the covalent organic framework. The broad signal decays as a function of time under constant illumination, while the narrower signal remains at an approximately constant level over the measurement timescale of 50 minutes.

The EPR measurement on the CO_2-saturated solution show a contribution of the broad signal centred at $g = 1.9927$ also observed under nitrogen atmosphere under illumination. The decay of this signal in CO_2-atmosphere is faster compared to that observed under nitrogen. The signal-to-noise ratio is not sufficient to unequivocally determine whether a small contribution of the narrower signal observed in nitrogen atmosphere is also present.

Table S1. DMPO spin adduct parameters used for EPR simulations.

**DMPO adduct**	***g*-value**	A_{14N}	A_{1H}	**Linewidth ([Gaussian Lorentzian])**
DMPO-CO_2^-	2.0054	41.6 MHz (14.8 G)	50.7 MHz (18.1 G)	[0.14 0.12] mT
DMPO-CR	2.0055	41.7 MHz (14.9 G)	55.5 MHz (19.8 G)	[0.30 0.26] mT
DMPO-OR_1	2.0056	37.4 MHz (13.4 G)	23.8 MHz (8.5 G)	[0.27 0.25] mT
DMPO-OR_2	2.0056	36.1 MHz (12.9 G)	29.0, 3.8 MHz (10.3, 1.4 G)	[0.09 0.12] mT
$DMPO_{degr,1}$	2.0065	19.6 MHz (7.0 G)	19.6 MHz (7.0 G)	[0.07 0.06] mT
$DMPO_{degr,2}$	2.0065	19.6 MHz (7.0 G)	–	[0.07 0.06] mT

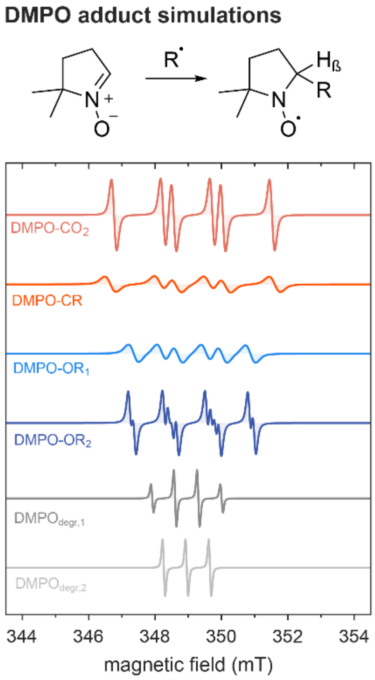

Fig.S11. Simulated EPR spectra of different DMPO adducts observed in the experimental measurements, the corresponding simulation parameters are listed in **Table S1**.

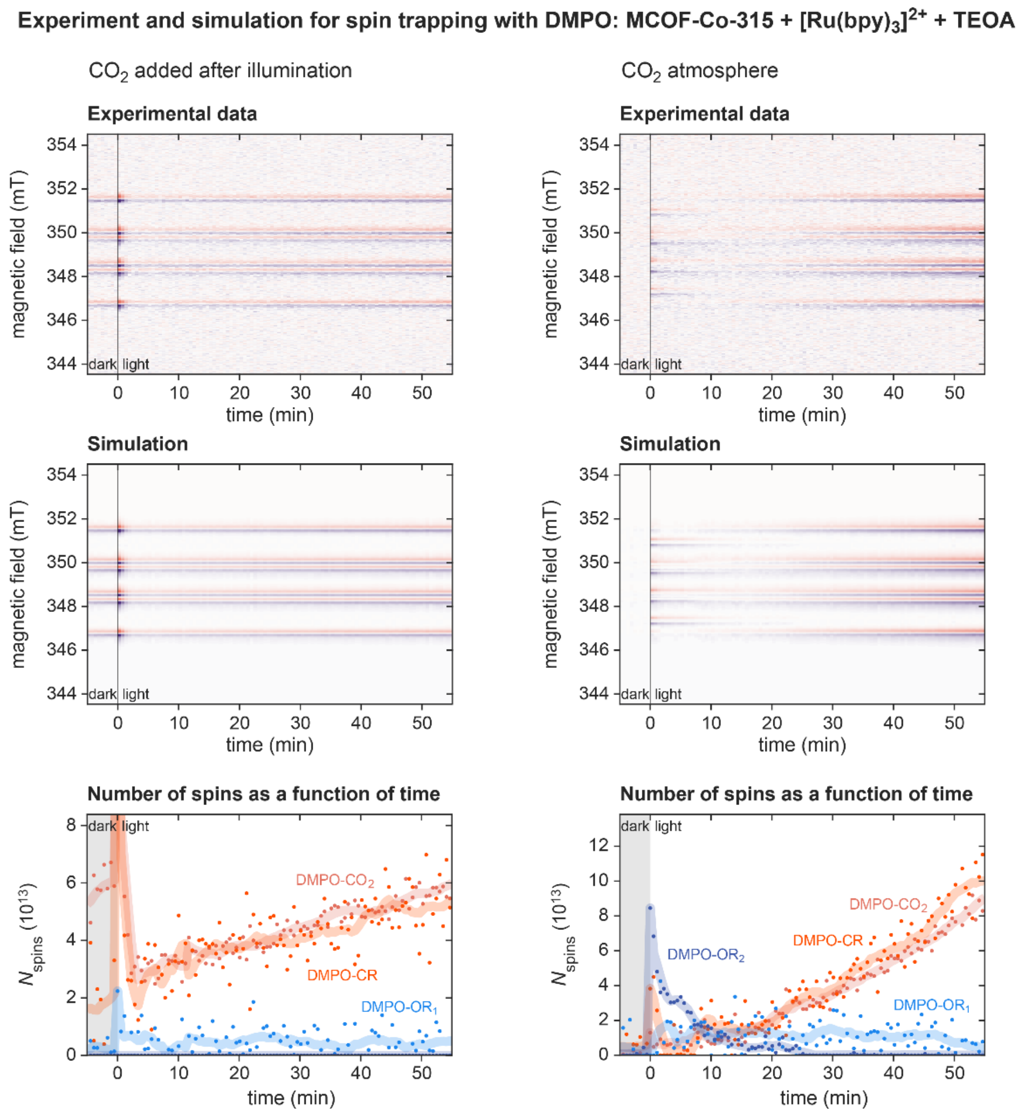

Fig.S12. EPR measurements performed at room temperature as a function of time prior to and during illumination with white light on solutions of MCOF-Co-315 with $[Ru(bpy)_3]^{2+}$ and TEOA in acetonitrile with the DMPO spin trap (red – negative, blue – positive). Experimental results are compared to simulations based on linear combinations of the contributions of different DMPO adducts, the corresponding extracted spin concentrations are shown as a function of time in the bottom panels.

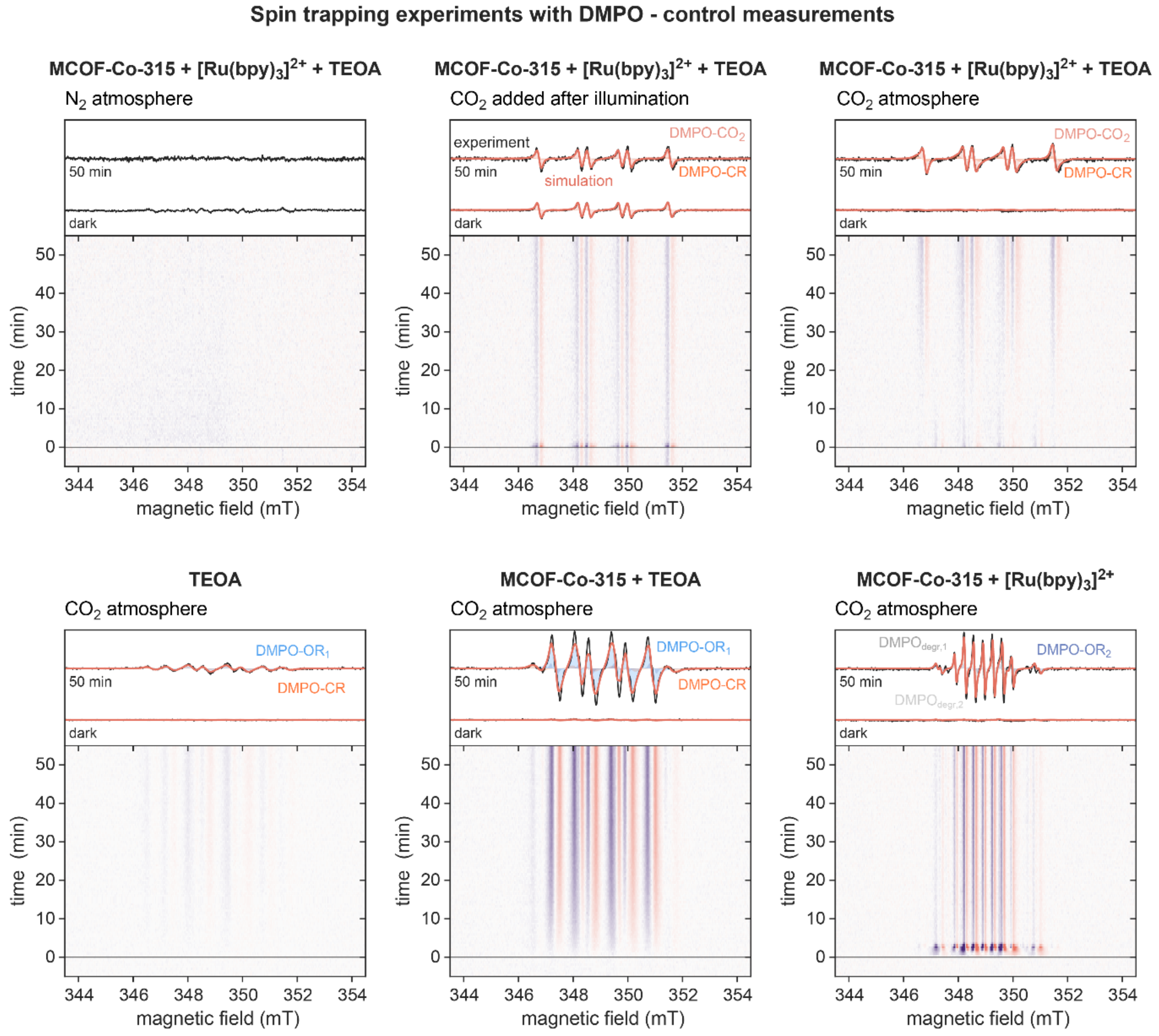

Fig.S13. EPR measurements performed at room temperature as a function of time prior to and during illumination with white light on solutions of MCOF-Co-315 with $[Ru(bpy)_3]^{2+}$ and TEOA in acetonitrile with the DMPO spin trap in nitrogen atmosphere, with CO_2 added after illumination and with CO_2 added prior to illumination. These results are compared to control measurements performed on a solution just containing TEOA in acetonitrile, a solution just containing MCOF-Co-315 and TEOA (in the absence of $[Ru(bpy)_3]^{2+}$) and a solution just containing MCOF-Co-315 and $[Ru(bpy)_3]^{2+}$ (in the absence of TEOA). Spectra extracted prior to illumination and after 50 minutes of illumination are compared to simulations performed as linear combination of the spectra of different DMPO adducts.

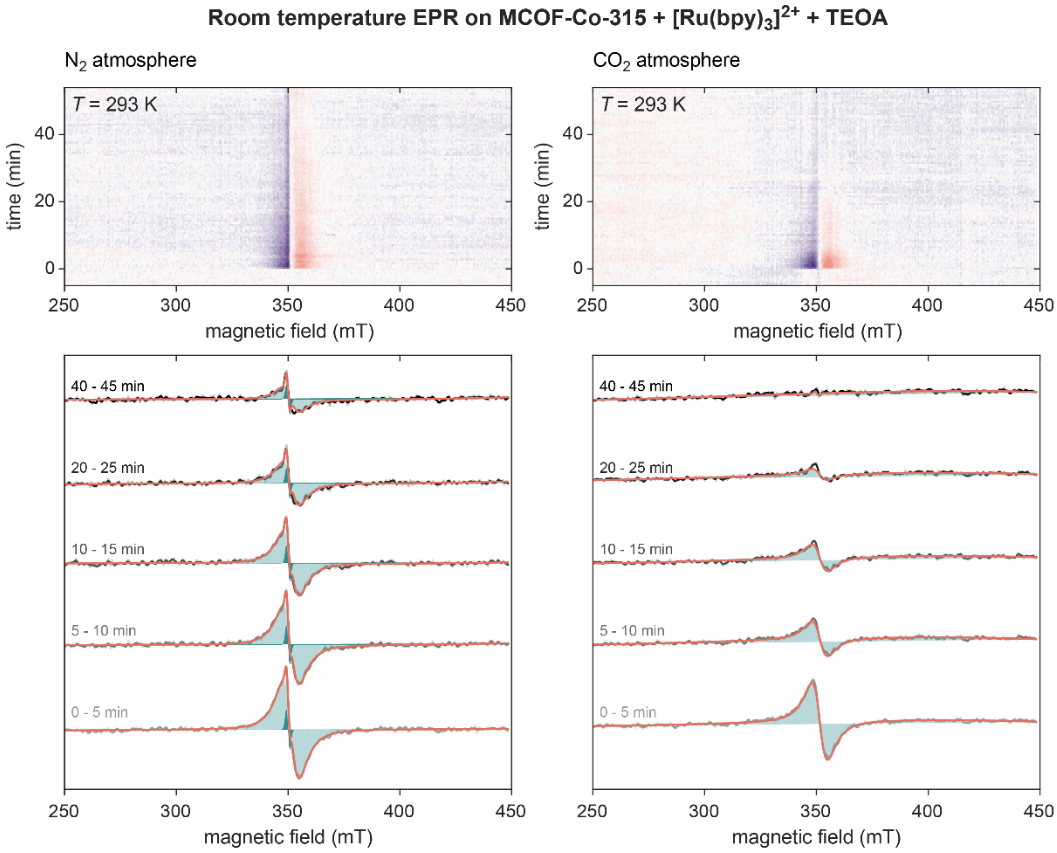

Fig.S14. EPR measurements performed at room temperature as a function of time prior to and during illumination with white light on solutions of MCOF-Co-315 with $[Ru(bpy)_3]^{2+}$ and TEOA in acetonitrile in nitrogen and CO_2 atmosphere. The top panels show the EPR spectra recorded as a function of time (red – negative, blue – positive), the bottom panels compare the extracted experimental spectra (grey) to simulations performed as a sum of two different contributions (see text for details).

6.3 Low temperature EPR measurements

In order to identify and characterise contributions due to Co species, EPR measurements were performed at cryogenic temperatures on solutions containing MCOF-Co-315, $[Ru(bpy)_3]^{2+}$ and TEOA in an acetonitrile:toluene 1:1 mixture saturated with CO_2 (solvent mixture selected to give a glass when frozen, photocatalytic activity of MCOF-Co-315 in this solvent mixture was verified at room temperature).

Co(II) (d^7) can be present in either a high-spin S = 3/2 or a low-spin S = 1/2 form, depending on the molecular environment, with distinctly different EPR spectra.[13] Low-spin Co(II) usually leads to signals with g-values close to 2, whereas the appearance of high-spin Co(II) EPR spectra is determined by the zero-field splitting interaction, which typically far exceeds the microwave quantum at X-band (9.4 GHz), leading to contributions of only transitions in the lower Kramers doublet ($m_S = \pm 1/2$ for D>0) and allowing modelling in terms of an effective S = 1/2 system with effective g-values often deviating significantly from 2 and determined by the rhombicity of the zero-field splitting interaction characterised by the ratio E/D ($0<E/D<1/3$, with E/D = 0 corresponding to axial symmetry and E/D = 1/3 to a maximally orthorhombic system).[14]

The Co(II) EPR spectrum obtained for MCOF-Co-315 could be simulated as an effective S = 1/2 system with $g_{\mathrm{eff},x}$ = 5.04, $g_{\mathrm{eff,y}}$ = 3.59, $g_{\mathrm{eff},z}$ = 1.95 with significant g-strain to reproduce the extensive broadening observed in the experimental spectrum, or equivalently as an S = 3/2 system with D > 200 GHz, g = 2.28 and E/D = 0.17 with D- and E-strain. The signal intensity of the EPR spectrum decreases significantly between 10 K and 20 K. The EPR results therefore unequivocally indicate the presence of high-spin Co(II) in MCOF-Co-315. As discussed in the main text, illumination of the sample leads to a reversible decrease in intensity of the observed Co(II) EPR signal, suggesting formation of EPR-silent Co(III) for a fraction of the cobalt centres.

EPR measurements were also performed on the precursor Co complex $Co(dabpy)_3$, where the EPR spectrum contains contributions from both a low-spin Co(II) centre, with a signal centred at $g \approx 2.1$ with eight clearly resolved peaks attributed to the hyperfine splitting due to the ^{59}Co (I = 7/2) nucleus ($g_\perp$ = 2.165 and $g_{||}$ = 2.172, $A(^{59}Co)_\perp$ = 264 MHz and $A(^{59}Co)_{||}$ = 2.172 MHz, 0.8% of the total signal), and a high-spin Co(II) centre in an almost perfectly axially symmetric environment, which can be simulated as either an effective S = 1/2 system with $g_{\mathrm{eff},x}$ = 5.03, $g_{\mathrm{eff},y}$ = 4.70, $g_{\mathrm{eff},z}$ = 2.44, or equivalently as an S = 3/2 system with D > 200 GHz, g = 2.43 and E/D = 0.01. Comparison of the spectra and simulation parameters for Co(II) in the precursor and MCOF-Co-315 indicate an environment with lower symmetry and increased heterogeneity in the latter. The low-temperature EPR spectrum of MCOF-Co-315 additionally contains a small sharp signal at $g \approx 2$ already observed at room temperature and attributed to a defect or charged state on the covalent organic framework.

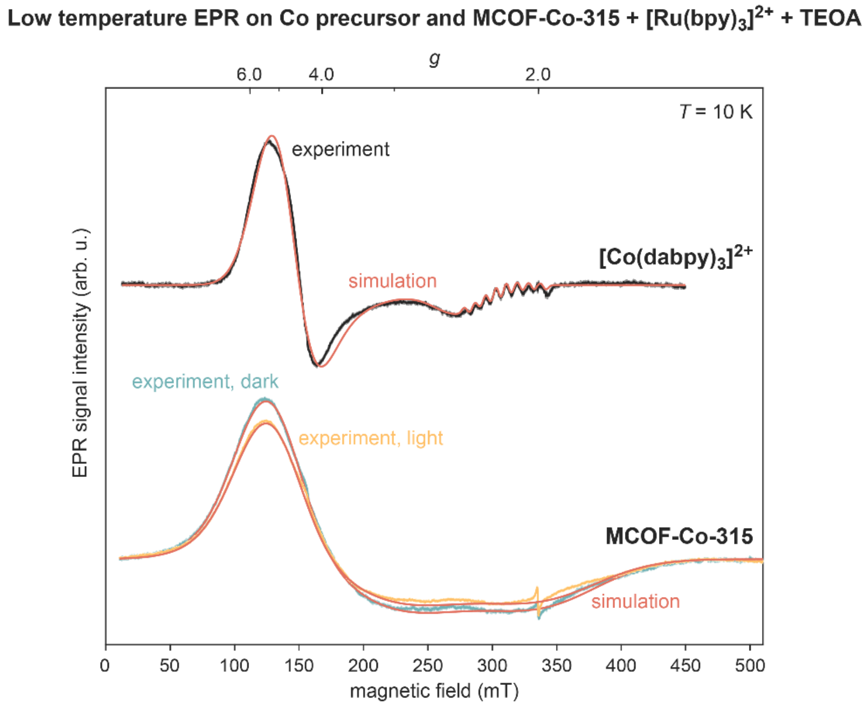

Fig.S15. Comparison of low-temperature EPR spectra recorded for the $[Co(dabpy)_3]^{2+}$ precursor complex and for MCOF-Co-315, $[Ru(bpy)_3]^{2+}$ and TEOA with simulations of the high-spin Co(II) signals giving rise to the broad contributions as effective $S = ½$ spin systems and of a small contribution of low-spin Co(II) in the case of the precursor (see text for simulation parameters).

7. Synchrotron FTIR Micro-spectroscopy

In situ gas-loaded synchrotron FTIR Micro-spectroscopy was carried out at the Multimode InfraRed Imaging and Microspectroscopy (MIRIAM) beamline at the Diamond Light Source, Harwell Science Campus (UK). The instrument is comprised of a Bruker Hyperion 3000 microscope in transmission mode with a 15× objective and condenser, and a liquid N_2 cooled MCT detector (mid-band, 50 um element), coupled to a Bruker Vertex 80V Fourier Transform IR interferometer using radiation generated from a bending magnet source. Spectra were collected (512 scans) in the range 600–4000 cm^{-1} at 4 cm^{-1} resolution and infrared spot size at the sample of approximately 30 × 30 μm. Samples were placed onto a zinc selenide (ZnSe) disk and placed within a Linkam FTIR 600 gas-tight sample cell, which was equipped with ZnSe windows, a heating stage and gas inlet and outlets. CO_2 was dosed volumetrically into the sample cell using mass flow controllers, the total flow rate being maintained at 100 cm^3 min^{-1} for all experiments. The gases were directly vented to an exhaust system and the total pressure in the cell was maintained at 1 bar for all experiments. The sample was desolvated under a flow of dry N_2 at 100 cm^3 min^{-1} and 393 K for 2 hours, and then cooled to room temperature under a continuous flow of N_2. For all single crystal samples, the initial gas flow was pure N_2 at a flow rate 100 cm^3 min^{-1}, which was then switched to 2%, 5%, 10%, 15%, 20% of CO_2 diluted in dry N_2. The sample is then regenerated in dry N_2 gas flow at room temperature.

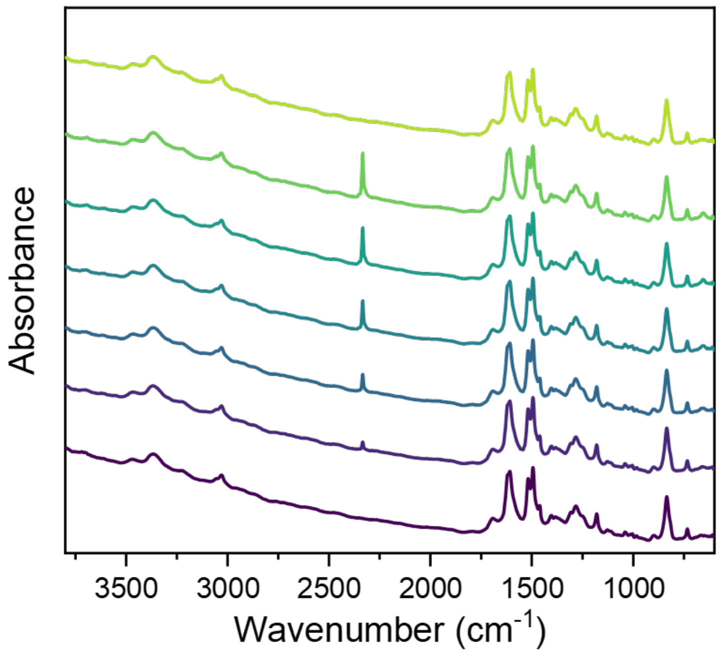

Fig.S16. Full range of in-situ synchrotron FT-IR spectroscopy of MCOF-Co-315 with increment CO_2 concentration in gas stream.

8. Catalytic Performance in Literature

Table S2. Summary of photocatalytic performance of COFs in literature for CO_2 conversion to CO.

Photocatalyst	Photosensitiser	Light condition	Sacrificial reagent	CO evolution rate (μmol g^{-1} h^{-1})	AQY (%)	Ref
Co-2,3-DHTA-COF	$[Ru(bpy)_3]Cl_2\cdot 6H_2O$	300 W Xe lamp (Cut 420 nm)	TEOA	18000	0.47 (450nm)	15
EPCo-COF-AT	$[Ru(bpy)_3]Cl_2\cdot 6H_2O$	300 W Xe lamp (Cut 420 nm)	TEOA	17700	0.49 (450nm)	16
COF-367-Co-NSs	$[Ru(bpy)_3]Cl_2\cdot 6H_2O$	300 W Xe lamp (Cut 420 nm)	Ascorbic acid	10162	/	17
CoPor-DPP-COF	$[Ru(bpy)_3]Cl_2\cdot 6H_2O$	300 W Xe lamp	TIPA[a]	10200	/	18
POSS-NiTFPP-COF-sqc	$[Ru(bpy)_3]Cl_2\cdot 6H_2O$, 2,2'-bipyridine	300 W Xe lamp (Cut 420 nm)	TIPA	9680	0.95 (420nm)	19
Co-PyPor-COF	$[Ru(bpy)_3]Cl_2\cdot 6H_2O$	300 W Xe lamp (Cut 420 nm)	TEOA	9645	/	20
GO-COF-366-Co	$[Ru(phen)_3](PF_6)_2$	300 W Xe lamp (Cut 320 nm)	TEOA	6525	/	21
Co10-COF	$[Ru(bpy)_3]Cl_2\cdot 6H_2O$	300 W Xe lamp (Cut 420 nm)	TEOA	4232	3.37 (420nm)	22
COF-366-Co/UiO-66-NH_2	$[Ru(bpy)_3]Cl_2\cdot 6H_2O$	300 W Xe lamp	TEOA	4092	2.40 (400nm)	23
H-COF-Ni	$[Ru(bpy)_3]Cl_2\cdot 6H_2O$, 2'2-bipyridine	300 W Xe lamp (Cut 420 nm)	TEOA	2847	/	24
CoNi-COF-3	$[Ru(bpy)_3]Cl_2\cdot 6H_2O$	300 W Xe lamp (Cut 420 nm)	TEOA	2567	2.95 (450nm)	25
CoP-TPE-COF	$[Ru(bpy)_3]Cl_2\cdot 6H_2O$	300 W Xe lamp (Cut 420 nm)	TEOA	2414	/	26
FBP-COF	2'2-bipyridine, $CoCl_2$	300 W Xe lamp (Cut 420 nm)	TEOA	2080	/	27
Co-FPy-CON	$(Ir[dF(CF_3)ppy]_2(dtbpy)PF_6$	300 W Xe lamp (Cut 420 nm)	TEOA	1683	6.6 (420nm)	28

**MCOF-Co-315**	**$[Ru(bpy)_3]Cl_2\cdot 6H_2O$**	**300 W Xe lamp (Cut 370 nm)**	**TEOA**	**1616**	**9.13 (450nm)**	*****
JUC-640-Co	$[Ru(bpy)_3]Cl_2\cdot 6H_2O$	300 W Xe lamp (Cut 380 nm)	BIH[b]	1510	1.48 (450nm)	29
TFBD-COF-Co-SA	$[Ru(bpy)_3]Cl_2\cdot 6H_2O$	300 W Xe lamp (Cut 400 nm)	TEOA	1480	/	30
Ni@TPHH-COF	$[Ru(bpy)_3]Cl_2\cdot 6H_2O$	300 W Xe lamp (Cut 420 nm)	TEOA	1270	3.96 (420nm)	31
H_2PReBpy-COF	/	300 W Xe lamp (Cut 400 nm)	TEA	1200	/	32
COF-366-Co(H)/Au	/	300 W Xe lamp (Cut 420 nm)	TEOA	1200	0.50 (420nm)	33
Re-Bpy-sp^2c-COF	/	300 W Xe lamp (Cut 420 nm)	TEOA	1040	0.50 (420nm)	34
DQTP COF-Co	$[Ru(bpy)_3]Cl_2\cdot 6H_2O$	300 W Xe lamp (Cut 420 nm)	TEOA	1020	/	35
Ni-TpBpy	$[Ru(bpy)_3]Cl_2\cdot 6H_2O$, 2’2-bipyridine	300 W Xe lamp (Cut 420 nm)	TEOA	1011	0.3 (420nm)	36
1D-PyTTA-COF	/	300 W Xe lamp (Cut 420 nm)	BIH	1003	0.04 (420nm)	37
Fe SAS/Tr-COF	$[Ru(bpy)_3]Cl_2\cdot 6H_2O$	300 W Xe lamp (Cut 420 nm)	TEOA	980	3.17 (420nm)	38
TPPD-COF	$[Ru(bpy)_3]Cl_2\cdot 6H_2O$	300 W Xe lamp (Cut 420 nm)	TEOA	951	/	39
BTTHz-1	$CoCl_2$	300 W Xe lamp (Cut 420 nm)	TEOA	774	8.6 (420nm)	40
$Ni(bpy)_3$@BtE-COF	$Ni(ClO_4)_2$, 2’2-bipyridine	300 W Xe lamp (Cut 420 nm)	TEOA	715	/	41
C3N4 (NH)/COF	2’2-bipyridine, $CoCl_2$	300 W Xe lamp (Cut 400 nm)	TEOA	562	/	42
COF-RuBpy-Co	$[Ru(bpy)_3]Cl_2\cdot 6H_2O$	300 W Xe lamp (Cut 420 nm)	TEOA	547	/	43

NiP-TPE-COF	$[Ru(bpy)_3]Cl_2 \cdot 6H_2O$	300 W Xe lamp (Cut 420 nm)	TEOA	525	/	44
CdS@COD	/	300 W Xe lamp (Cut 420 nm)	BIH	507	0.21 (420nm)	45
Ni-PCD@TD-COF	$[Ru(bpy)_3]Cl_2 \cdot 6H_2O$	300 W Xe lamp (Cut 420 nm)	TEOA	480	0.31 (420nm)	46
PI-COFs	$Ni(ClO_4)_2 \cdot 6H_2O$, 2'2-bipyridine	300 W Xe lamp (Cut 420 nm)	TEOA	480	0.55 (380nm)	47
BTE-TBD-COF	/	300 W Xe lamp	/	382	0.11 (405nm)	48
COF-5/CoAl-LDH	/	300 W Xe lamp (Cut 420 nm)	/	265	/	49
TT-COF	/	300 W Xe lamp (Cut 420 nm)	TEA	248	0.25 (500nm)	50
Cu-COF	/	300 W Xe lamp (Cut 420 nm)	TEOA	206	/	51
rGO_{15}@TpPa1	2'2-bipyridine, $CoCl_2$	300 W Xe lamp (Cut 420 nm)	TEOA	200	0.50 (420nm)	52
NH_2-T-COF	/	300 W Xe lamp	Ascorbic acid	188	6.81 (365nm)	53
BPDA-COF-TiO_2	/	150 W Xe lamp	BIH	91	/	54
Ga_2O_3/COF	/	300 W Xe lamp (Cut 420 nm)	/	86	/	55
COF-318/TNF-15	/	300 W Xe lamp (Cut 420 nm)	/	70	/	56
DhaTph-Cu	/	300 W Xe lamp	/	16	/	57
T-101/COF	2'2-bipyridine, $CoCl_2$	300 W Xe lamp (Cut 420 nm)	TEOA	12	/	58
COF-366-Co	$[Ru(bpy)_3]Cl_2 \cdot 6H_2O$	300 W Xe lamp (Cut 400 nm)	TEOA	10	/	59
NAHN-Tp COF	[Ir-ppy]	300 W Xe lamp (Cut 420 nm)	TEOA	9	/	60

COF-366-Fe@CeO_2	/	300 W Xe lamp (Cut 420 nm)	/	8	/	61
2D CN-COF	/	300 W Xe lamp (Cut 420 nm)	/	7	0.03 (420nm)	62
COF-TVBT-PA	/	300 W Xe lamp	Ascorbic acid	7	/	63

a TIPA : triisopropanolamine

b BIH : 1,3-dimethyl-2-phenyl-2,3-dihydro-1H-benzo[d]imidazole

* This Work

9. Single Crystal X-Ray Diffraction of $[Co^{II}(dabpy)_3]Cl_2$

Single crystal X-ray diffraction data (λ = 1.54180 Å) for $[Co^{II}(dabpy)_3]Cl_2$ was collected on a Rigaku Oxford Diffraction SuperNova, a diffractometer with an Oxford CryoSystems CryoStream (150 K).[64] Data reduction was performed using CrysAlisPro, the structure solved with ShelXT[65], and refined using the CRYSTALS suite[66-68]. Additional details are provided in the ESI (CIF). Crystallographic data have been deposited with the Cambridge Crystallographic Data Centre (CCDC 2420179) and are available at www.ccdc.cam.ac.uk/data_request/cif.

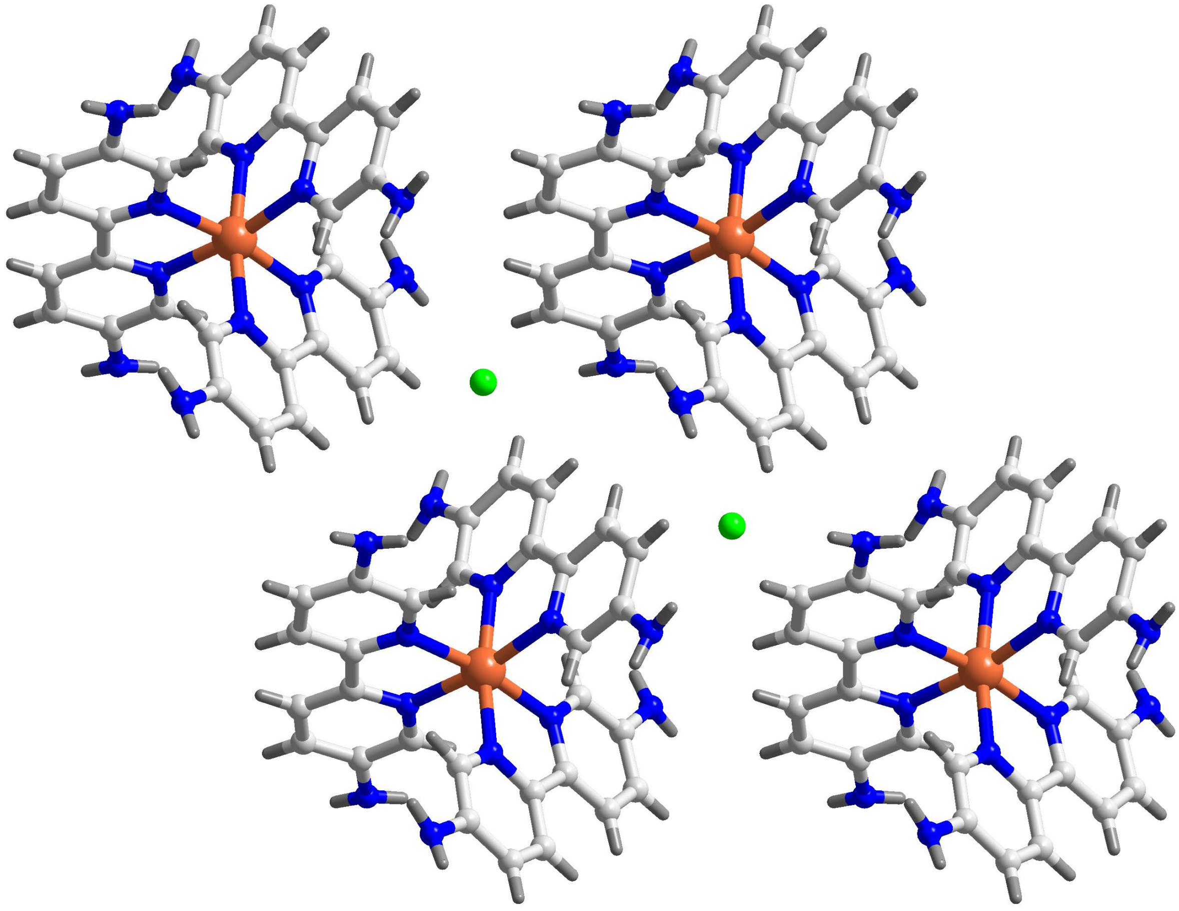

Fig.S17. Structure of $[Co^{II}(dabpy)_3]Cl_2$ Viewed from *c* axis. (C, silver; H, grey; Co, oprange; N, blue; Cl, green.)

Table S3. Single Crystal Data of $[Co^{II}(dabpy)_3]Cl_2$.

	$[Co^{II}(dabpy)_3]Cl_2$
Formula	$C_{30}H_{30}ClCoN_{12}$
Formula Weight	653.04
Crystal System	Hexagonal
Space Group	P -6 2 c
a (Å)	10.848(1)
b (Å)	10.848(1)
c (Å)	23.101(1)
Volume (Å^3)	2354.3(1)
Z	2
R_1 (%)	3.31
wR_2 (%)	9.51
R_{int} (%)	3.90
GoF	1.012
CCDC number	2420179

10. Extended X-ray Absorption Fine Structure Analysis

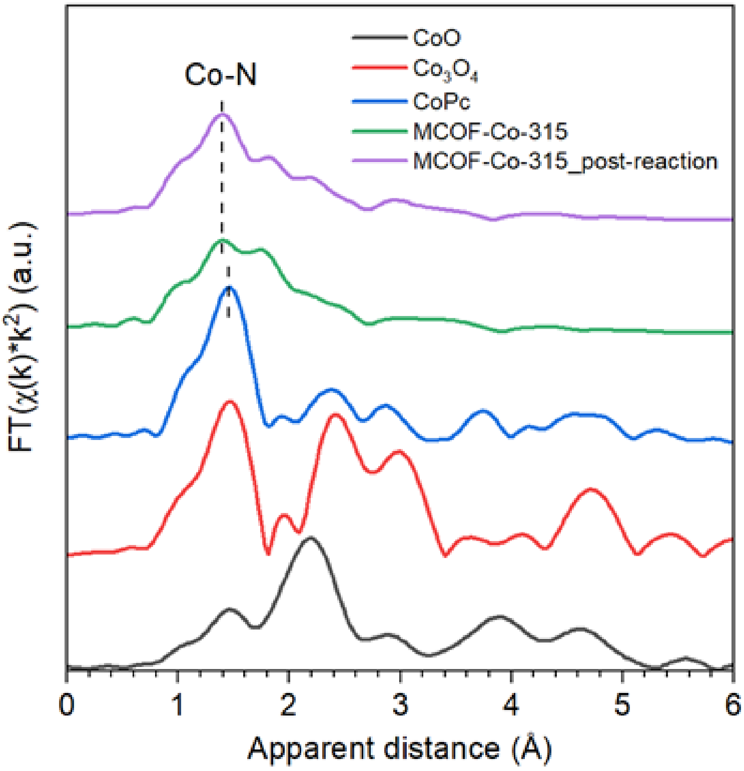

Fig.S18. Fourier transforms of k^2-weighted Co K-edge EXAFS spectra for MCOF-Co-315, MCOF-Co-315_ post-reaction, and reference samples (CoO, Co_3O_4, and CoPc).

X-ray absorption spectroscopy (XAS) was conducted to examine changes in the chemical and coordination environment of Co atoms in MCOF-Co-315 before and after photocatalysis. The X-ray absorption near-edge structure (XANES) analysis at the Co K-edge reveals that the absorption edge of MCOF-Co-315 after the reaction (MCOF-Co-315_post-reaction) shifts to a higher energy compared to that before the reaction. This shift indicates a slight increase in the average valence state of Co atoms (**Fig.5b**). Extended X-ray absorption fine structure (EXAFS) analysis was performed to investigate the coordination environment of Co atoms. The Fourier-transformed EXAFS (FT-EXAFS) spectra of the MCOF-Co-315 (**Fig.S18**) reveal a prominent peak around 1.40 Å, corresponding to the Co-N bond, which closely matches the Co-N peak observed in the CoPc reference. The enhanced intensity of the Co-N peak in MCOF-Co-315_post-reaction suggests a strengthened Co-N interaction, likely due to oxidation or structural reorganization following the reaction. This observation is consistent with the results from XANES and EPR measurements.

11. Reference

[1] Björemark, P. M.; Jönsson, J.; Håkansson, M. Absolute Asymmetric Synthesis: Viedma Ripening of $[Co(bpy)_3]^{2+}$ and Solvent-Free Oxidation to $[Co(bpy)_3]^{3+}$. *Chem. Eur. J.* **2015**, *21*, 10630–10633.

[2] Willems, T. F., Rycroft, C. H., Kazi, M., Meza, J. C., Haranczyk, M. Algorithms and tools for high-throughput geometry-based analysis of crystalline porous materials. *Microporous Mesoporous Mater.* **2012**, *149*, 134–141.

[3] Anderson, R., Gómez-Gualdrón, D. A. Increasing topological diversity during computational "synthesis" of porous crystals: how and why. *CrystEngComm*, **2019**, *21*, 1653–1665.

[4] Turcani, L., Berardo, E., Jelfs, K. E. stk: A python toolkit for supramolecular assembly. *J. Comput. Chem.* **2018**, *39*, 1931–1942.

[5] Lu, M., Zhang, S., Yang, M., Liu, Y., Liao, J., Huang, P., Zhang, M., Li, S., Su, Z., Lan, Y. Dual Photosensitizer Coupled Three-Dimensional Metal-Covalent Organic Frameworks for Efficient Photocatalytic Reactions. *Angew. Chem. Int. Ed.* **2023**, *62*, e202307632.

[6] Macrae, C. F., Sovago, I., Cottrell, S. J., Galek, P. T. A., McCabe, P., Pidcock, E., Platings, M., Shields, G. P., Stevens, J. S., Towler, M., Wood, P. A. Mercury 4.0: from visualization to analysis, design and prediction. *J. Appl. Crystallogr.* **2020**, *53*, 226–235.

[7] Kühne, T. D., Iannuzzi, M., Del Ben, M., Rybkin, V. V., Seewald, P., Stein, F., Laino, T., Khaliullin, R. Z., Schütt, O., Schiffmann, F., Golze, D., Wilhelm, J., Chulkov, S., Bani-Hashemian, M. H., Weber, V., Borštnik, U., Taillefumier, M., Jakobovits, A. S., Lazzaro, A., Pabst, H., Müller, T., Schade, R., Guidon, M., Andermatt, S., Holmberg, N., Schenter, G. K., Hehn, A., Bussy, A., Belleflamme, F., Tabacchi, G., Glöß, A., Lass, M., Bethune, I., Mundy, C. J., Plessl, C., Watkins, M., VandeVondele, J., Krack, M., Hutter, J. CP2K: An electronic structure and molecular dynamics software package - Quickstep: Efficient and accurate electronic structure calculations. *J Chem Phys.* **2020**, *152*, 194103.

[8] Perdew, J. P., Burke, K., Ernzerhof, M. Generalized Gradient Approximation Made Simple. *Phys. Rev. Lett.* **1996**, *77*, 3865–3868.

[9] VandeVondele, J., Hutter, J. Gaussian basis sets for accurate calculations on molecular systems in gas and condensed phases. *J Chem Phys.* **2007**, *127*, 114105.

[10] Wittmann, J. J., Can, T. V., Eckardt, M., Harneit, W., Griffin, R. G., Corzilius, B. High-precision measurement of the electron spin g factor of trapped atomic nitrogen in the endohedral fullerene N@C60. *J. Magn. Reson.* **2018**, *290*, 12–17.

[11] Stoll, S., Schweiger, A. EasySpin, a comprehensive software package for spectral simulation and analysis in EPR. *J. Magn. Reson.* **2006**, *178*, 42–55.

[12] Buettner, G. R. Spin Trapping - Electron-Spin-Resonance Parameters of Spin Adducts. *Free Radic. Bio. Med.* **1987**, *3*, 259–303.

[13] Abragam, A., Bleaney, B. *Electron Paramagnetic Resonance of Transition Ions*. (Clarendon Press, 1970).

[14] Pilbrow, J. R. Effective g Values for S = 3/2 and S = 5/2. *J. Magn. Reson.* **1978**, *31*, 479–490.

[15] Zhang, Q., Gao, S., Guo, Y., Wang, H., Wei, J., Su, X., Zhang, H., Liu, Z., Wang, J. Designing covalent organic frameworks with Co-O_4 atomic sites for efficient CO_2 photoreduction. *Nat. Commun.* **2023**, *14*, 1147.

[16] Lin, W., Lin, F., Lin, J., Xiao, Z., Yuan, D., Wang, Y. Efficient Photocatalytic CO_2 Reduction in Ellagic Acid–Based Covalent Organic Frameworks. *J. Am. Chem. Soc.* **2024**, *146*, 16229–16236.

[17] Liu, W., Li, X., Wang, C., Pan, H., Liu, W., Wang, K., Zeng, Q., Jiang, J. A scalable general synthetic approach toward ultrathin imine-linked two-dimensional covalent organic framework nanosheets for photocatalytic CO_2 reduction. *J. Am. Chem. Soc.* **2019**, *141*, 17431–17440.

[18] Wang, X., Ding, X., Wang, T., Wang, K., Jin, Y., Han, Y., Zhang, P., Li, N., Wang, H., Jiang, J. Two-dimensional porphyrin-based covalent organic framework with enlarged inter-layer spacing for tunable photocatalytic CO_2 reduction. *ACS Appl. Mater. Interfaces* **2022**, *14*, 41122–41130.

[19] Gao, Y., Li, S., Gong, L., Li, J., Qi, D., Liu, N., Bian, Y., Jiang, J. Unprecedented POSS-Linked 3D Covalent Organic Frameworks with 2-Fold Interpenetrated scu or sqc Topology Regulated by Porphyrin Center for Photocatalytic CO_2 Reduction. *Angew. Chem. Int. Ed.* **2024**, *63*, e202404156.

[20] Luan, T. X., Wang, J. R., Li, K., Li, H., Nan, F., Yu, W. W., Li, P. Z. Highly Enhancing CO_2 Photoreduction by Metallization of an Imidazole‐linked Robust Covalent Organic Framework. *Small* **2023**, *19*, 2303324.

[21] Gong, Y. N., Mei, J. H., Shi, W. J., Liu, J. W., Zhong, D. C., Lu, T. B. Boosting CO_2 Photoreduction to Formate or CO with High Selectivity over a Covalent Organic Framework Covalently Anchored on Graphene Oxide. *Angew. Chem. Int. Ed.* **2024**, *63*, e202318735.

[22] Dong, S., Tan, Z., Chen, Q., Huang, G., Wu, L., Bi, J. Cobalt quantum dots as electron collectors in ultra-narrow bandgap dioxin linked covalent organic frameworks for boosting photocatalytic solar-to-fuel conversion. *J. Colloid Interface Sci* **2022**, *628*, 573–582.

[23] Quach, T. A., Gopalakrishnan, V. N., Becerra, J., Nguyen, D. T., Ahad, J. M., Mohan, S., Do, T. O. Z-scheme heterojunction of chemically integrated COF-366-Co/UiO-66-NH_2 MOFs nanocomposites for selective production of CO via CO_2 solar-drive photoreduction. *Catal. Today* **2023**, *421*, 114218.

[24] Yang, S., Sa, R., Zhong, H., Lv, H., Yuan, D., Wang, R. Microenvironments enabled by covalent organic framework linkages for modulating active metal species in photocatalytic CO_2 reduction. *Adv. Funct. Mater.* **2022**, *32*, 2110694.

[25] Wang, J., Zhu, W., Meng, F., Bai, G., Zhang, Q., Lan, X. Integrating Dual-Metal Sites into Covalent Organic Frameworks for Enhanced Photocatalytic CO_2 Reduction. *ACS Catal.* **2023**, *13*, 4316–4329.

[26] Lv, H., Sa, R., Li, P., Yuan, D., Wang, X., Wang, R. Metalloporphyrin-based covalent organic frameworks composed of the electron donor-acceptor dyads for visible-light-driven selective CO_2 reduction. *Sci China Chem* **2020**, *63*, 1289–1294.

[27] Fu, Z., Shu, C., Wang, X., Chen, L., Wang, X., Liu, L., Wang, K., Clowes, R., Chong, S. Y., Wu, X., Cooper, A. I. Fluorinated covalent organic frameworks coupled with molecular cobalt cocatalysts for efficient photocatalytic CO_2 reduction. *CCS Chemistry* **2023**, *5*, 2290–2300.

[28] Wang, X., Fu, Z., Zheng, L., Zhao, C., Wang, X., Chong, S. Y., McBride, F., Raval, R., Bilton, M., Liu, L., Wu, X., Chen, L., Sprick, R., Cooper, A. I. Covalent organic framework nanosheets embedding single cobalt sites for photocatalytic reduction of carbon dioxide. *Chem. Mater.* **2020**, *32*, 9107–9114.

[29] Ding, J., Guan, X., Lv, J., Chen, X., Zhang, Y., Li, H., Zhang, D., Qiu, S., Jiang, H., Fang, Q. Three-dimensional covalent organic frameworks with ultra-large pores for highly efficient photocatalysis. *J. Am. Chem. Soc.* **2023**, *145*, 3248–3254.

[30] Yang, Y., Lu, Y., Zhang, H. Y., Wang, Y., Tang, H. L., Sun, X. J., Zhang, G., Zhang, F. M. Decoration of active sites in covalent–organic framework: An effective strategy of building efficient photocatalysis for CO_2 reduction. *ACS Sustainable Chem. Eng.* **2021**, *9*, 13376–13384.

[31] Dong, M. Zhou, J., Zhong, J., Li, H., Sun, C., Han, Y., Kou, J., Kang, Z., Wang, X., Su, Z. CO_2 dominated bifunctional catalytic sites for efficient industrial exhaust conversion. *Adv. Funct. Mater.* **2022**, *32*, 2110136.

[32] Song, D., Xu, W., Li, J., Zhao, J., Shi, Q., Li, F., Sun, X., Wang, N. "All-in-one" covalent organic framework for photocatalytic CO_2 reduction. Chin. J. Catal. **2022**, *43*, 2425–2433.

[33] Nair Gopalakrishnan, V., Quach, T. A., Becerra, J., Mohan, S., Ahad, J. M., Béland, F., Do, T. O. Au Nanoparticle-Anchored Hollow Nanospheres of a Single-Atomized Porphyrin-Covalent Organic Framework Hybrid for Boosting Photoreduction of CO_2 under Solar Irradiation. *J. Phys. Chem. C* **2023**, *127*, 7929–7937.

[34] Fu, Z., Wang, X., Gardner, A. M., Wang, X., Chong, S. Y., Neri, G., Cowan, A. J., Liu, X., Vogel, A., Clowes, R., Bilton, M., Chen, L., Sprick, R. S., Cooper, A. I. A stable covalent organic framework for photocatalytic carbon dioxide reduction. *Chem. Sci.* **2020**, *11*, 543–550.

[35] Lu, M., Li, Q., Liu, J., Zhang, F. M., Zhang, L., Wang, J. L., Kang, Z. H., Lan, Y. Q. Installing earth-abundant metal active centers to covalent organic frameworks for efficient heterogeneous photocatalytic CO_2 reduction. Appl. Catal. B: Environ. **2019**, *254*, 624–633.

[36] Zhong, W., Sa, R., Li, L., He, Y., Li, L., Bi, J., Zhuang, Z., Yu, Y., Zou, Z. A covalent organic framework bearing single Ni sites as a synergistic photocatalyst for selective photoreduction of CO_2 to CO. *J. Am. Chem. Soc.* **2019**, *141*, 7615–7621.

[37] Zou, L., Chen, Z. A., Si, D. H., Yang, S. L., Gao, W. Q., Wang, K., Huang, Y., Cao, R. Boosting CO2 Photoreduction via Regulating Charge Transfer Ability in a One-Dimensional Covalent Organic Framework. *Angew. Chem. Int. Ed.* **2023**, *62*, e202309820.

[38] Ran, L., Li, Z., Ran, B., Cao, J., Zhao, Y., Shao, T., Leung, M. J., Sun, L., Hou, J. Engineering single-atom active sites on covalent organic frameworks for boosting CO_2 photoreduction. *J. Am. Chem. Soc.* **2022**, *144*, 17097–17109.

[39] Dong, M., Li, W., Zhou, J., You, S. Q., Sun, C. Y., Yao, X. H., Qin, C., Wang, X. L., Su, Z. M. Microenvironment Modulation of Imine-Based Covalent Organic Frameworks for CO_2 Photoreduction. *Chin. J. Chem.* **2022**, *40*, 2678–2684.

[40] Tang, Q., Gu, Y. Y., Ning, J., Yan, Y., Shi, L., Zhou, M., Wei, H., Ren, X., Li, X., Wang, J., Tang, C., Hao, L., Ye, J. Boosting photocatalysis of hydrazone-linked covalent organic frameworks through introducing electron-rich conjugated aldehyde. Chem. Eng. J. **2023**, *470*, 144106.

[41] He, Y., Zhao, Y., Wang, X., Liu, Z., Yu, Y., Li, L. Multiple Heteroatom-Hydrogen Bonds Bridging Electron Transport in Covalent Organic Framework-Based Supramolecular System for Photoreduction of CO_2. *Angew. Chem. Int. Ed.* **2023**, *62*, e202307160.

[42] Wang, J. Yu, Y., Cui, J., Li, X., Zhang, Y., Wang, C., Yu, X., Ye, J. Defective g-C_3N_4/covalent organic framework van der Waals heterojunction toward highly efficient S-scheme CO_2 photoreduction. *Appl. Catal. B: Environ.* **2022**, *301*, 120814.

[43] Gong, L. J., Liu, L. Y., Zhao, S. S., Yang, S. L., Si, D. H., Wu, Q. J., Wu, Q., Huang, Y., Cao, R. Rapid charge transfer in covalent organic framework via through-bond for enhanced photocatalytic CO_2 reduction. *Chem. Eng. J.* **2023**, *458*, 141360.

[44] Lv, H., Sa, R., Li, P., Yuan, D., Wang, X., Wang, R. Metalloporphyrin-based covalent organic frameworks composed of the electron donor-acceptor dyads for visible-light-driven selective CO_2 reduction. *Sci. China Chem.* **2020**, *63*, 1289–1294.

[45] Zou, L., Sa, R., Zhong, H., Lv, H., Wang, X., Wang, R. Photoelectron transfer mediated by the interfacial electron effects for boosting visible-light-driven CO_2 reduction. *ACS Catal.* **2022**, *12*, 3550–3557.

[46] Zhong, H., Sa, R., Lv, H., Yang, S., Yuan, D., Wang, X., Wang, R. Covalent organic framework hosting metalloporphyrin-based carbon dots for visible-light-driven selective CO_2 reduction. *Adv. Funct. Mater.* **2020**, *30*, 2002654.

[47] Chen, X., Dang, Q., Sa, R., Li, L., Li, L., Bi, J., Zhang, Z., Long, J., Yu, Y., Zou, Z. Integrating single Ni sites into biomimetic networks of covalent organic frameworks for selective photoreduction of CO_2. *Chem. Sci.* **2020**, *11*, 6915–6922.

[48] Yang, L., Yan, W., Yang, N., Wang, G., Bi, Y., Tian, C., Liu, H., Zhu, X. Regulating π-Conjugation in sp^2-Carbon-Linked Covalent Organic Frameworks for Efficient Metal-Free CO_2 Photoreduction with H_2O. *Small*, **2023**, *19*, 2208118.

[49] Ou, S., Zhou, M., Chen, W., Zhang, Y., Liu, Y. COF-5/CoAl-LDH Nanocomposite Heterojunction for Enhanced Visible-Light-Driven CO_2 Reduction. *ChemSusChem*, **2022**, *15*, e202200184.

[50] Dey, A., Rahimi, F. A., Barman, S., Hazra, A., Maji, T. K. Metal-free 3D donor–acceptor COF with low exciton binding for solar fuel production based on CO_2 reduction. *J. Mater. Chem. A* **2023**, *11*, 13615–13622.

[51] Tu, W., Yang, Y., Chen, C., Zhou, T., Li, T., Wang, H., Wu, S., Zhou, Y., O'Hare, D., Zou Z., Xu, R. Cu–O/N Single Sites Incorporated 2D Covalent Organic Framework Ultrathin Nanobelts for Highly Selective Visible-Light-Driven CO_2 Reduction to CO. *Small Structures*, **2023**, *4*, 2200233.

[52] Gopalakrishnan, V. N. Nguyen, D., Becerra, J., Sakar, M., Ahad, J. M. E., Jautzy, J. J., Mindorff, L. M., Béland, F., Do, T. O. Manifestation of an enhanced photoreduction of CO_2 to CO over the in situ synthesized rGO–covalent organic framework under visible light irradiation. *ACS Appl. Energy. Mater.* **2021**, *4*, 6005–6014.

[53] Wang, Y., Hu, Z., Wang, W., Li, Y., He, H., Deng, L., Zhang, Y., Huang, J., Zhao, N., Yu, G., Liu, Y. N. Rational design of defect metal oxide/covalent organic frameworks Z-scheme heterojunction for photoreduction CO_2 to CO. *Appl. Catal. B: Environ.* **2023**, *327*, 122419.

[54] Putta Rangappa, A., Praveen Kumar, D., Do, K. H., Wang, J., Zhang, Y., Kim, T. K. Synthesis of Pore-Wall-Modified Stable COF/TiO_2 Heterostructures via Site-Specific Nucleation for an Enhanced Photoreduction of Carbon Dioxide. *Adv. Sci.* **2023**, *10*, 2300073.

[55] Yang, R., Chen, Q., Huang, G., Bi, J. Interfacial engineering of novel inorganic-organic β-Ga_2O_3/COF heterojunction for accelerated charge transfer towards artificial photosynthesis. *Environ. Res.* **2023**, *216*, 114541.

[56] Gao, Y., Tan, Z., Yang, R., Huang, G., Bi, J. Integrating polyarylether-COFs with TiO_2 nanofibers for enhanced visible-light-driven CO_2 reduction in artificial photosynthesis. *Appl. Surf. Sci.* **2022**, *605*, 154605.

[57] Zhang, Z., Lu, J., Yang, K., Cao, J., Zhao, Y., Ge, K., Wang, S., Yang, Y., Zhang, Y., Yang, Y. DhaTph Tubes and DhaTph-Cu Tubes with Hollow Tubular Structure and Their Photocatalytic Reduction of CO_2. *ChemistrySelect* **2022**, *7*, e202201203.

[58] An, X., Bian, J., Zhu, K., Liu, R., Liu, H., Qu, J. Facet-dependent activity of TiO_2/covalent organic framework S-scheme heterostructures for CO_2 photoreduction. Chem. Eng. J. **2022**, *442*, 135279.

[59] Zhao, S. S., Liang, J., Si, D. H., Mao, M. J., Huang, Y. B., Cao, R. Superheterojunction covalent organic frameworks: Supramolecular synergetic charge transfer for highly efficient photocatalytic CO_2 reduction. Appl. Catal. B: Environ. **2023**, *333*, 122782.

[60] You, S. Q., Zhou, J., Chen, M. M., Sun, C. Y., Qi, X. j., Yousaf, A., Wang, X. L., Su, Z. M. A hydrazone-based covalent organic framework/iridium (III) complex for photochemical CO_2 reduction with enhanced efficiency and durability. *J. Catal.* **2020**, *392*, 49–55.

[61] Li, S., Yu, H., Wang, Y., Wang, S., Zhang, L., Zhu, P., Gao, C., Yu, J. Engineering covalently integrated COF@CeO_2 Z-scheme heterostructure for visible light driven photocatalytic CO_2 conversion. *Appl. Surf. Sci.* **2023**, *615*, 156335.

[62] Song, X., Wu, Y., Zhang, X., Li, X., Zhu, Z., Ma, C., Yan, Y., Huo, P., Yang, G. Boosting charge carriers separation and migration efficiency via fabricating all organic van der Waals heterojunction for efficient photoreduction of CO_2. *Chem. Eng. J.* **2021**, *408*, 127292.

[63] Zhou, J., Cui, J. X., Dong, M., Sun, C. Y., You, S. Q., Wang, X. L., Zhou, Z. Y., Su, Z. M. Synergetic effect of H+ adsorption and ethylene functional groups of covalent organic frameworks on the CO_2 photoreduction in aqueous solution. *Chem. Commun.* **2020**, *56*, 7261–7264.

[64] Cosier, J. T., Glazer, A. M. A nitrogen-gas-stream cryostat for general X-ray diffraction studies. *J. Appl. Cryst.* **1986**, 19, 105–107.

[65] Sheldrick, G. M. SHELXT–Integrated space-group and crystal-structure determination. *Acta Cryst.* **2015**, 71, 3–8.

[66] Betteridge, P. W., Carruthers, J. R., Cooper, R. I., Prout, K., Watkin, D. J. CRYSTALS version 12: software for guided crystal structure analysis. *J. Appl. Cryst.* **2003**, 36, 1487–1487.

[67] Parois, P., Cooper, R. I., Thompson, A. L. *Chem. Cent. J.* **2015**, 9, 30.

[68] Cooper, R. I., Thompson, A. L., Watkin, D. J. CRYSTALS enhancements: dealing with hydrogen atoms in refinement. *J. Appl. Cryst.* **2010**, 43, 1100–1107.
